# Integrated apoptotic extracellular vesicle-recruitment peptide coating reprograms the diabetic bone microenvironment and orchestrates enhanced implant osseointegration

**DOI:** 10.1016/j.bioactmat.2026.05.059

**Published:** 2026-06-11

**Authors:** Jining Shen, Gaoran Ge, Tianpeng Xu, Qiufei Wang, Yi Qin, Xiaoheng Lu, Jin Liu, Pengcheng Xu, Kefan Wu, Fan Liu, Xing Yang, Dechun Geng, Yake Liu

**Affiliations:** a Department of Orthopaedics, Affiliated Hospital of Nantong University, Nantong, Jiangsu, 226001, China; b Department of Orthopaedics, The First Affiliated Hospital of Soochow University, No. 899 Pinghai Road, Suzhou, Jiangsu, 215006, China; c Department of Orthopedic, The Affiliated Suzhou Hospital of Nanjing Medical University, Suzhou Municipal Hospital, Gusu School of Nanjing Medical University, Suzhou, Jiangsu, 215008, China; d Department of Orthopedics, First People's Hospital of Changshu City, Shuyuan Street, Changshu, Jiangsu, 215500, China; e Department of Infectious Diseases, The Affiliated Infectious Diseases Hospital, Suzhou Medical College of Soochow University, The Fifth People's Hospital of Suzhou, Jiangsu, 215100, China; f Department of Rehabilitation Medicine, Suzhou Municipal Hospital, Nanjing Medical University Affiliated Suzhou Hospital, Suzhou, Jiangsu, 215008, China; g Orthopedics and Sports Medicine Center, Suzhou Municipal Hospital, Nanjing Medical University Affiliated Suzhou Hospital, Suzhou, Jiangsu, 215008, China; h Anesthesiology Department, Suzhou Municipal Hospital, Nanjing Medical University Affiliated Suzhou Hospital, Suzhou, Jiangsu, 215008, China

**Keywords:** Apoptotic extracellular vesicle, Diabetes mellitus, Osteoimmunomodulatory, Osseointegration, Mussel-inspired adhesion

## Abstract

Achieving stable prosthetic fixation remains a formidable challenge under diabetic conditions, primarily due to deficient osseointegration driven by impaired macrophage polarization and persistent inflammatory dysregulation within the bone microenvironment. To overcome these challenges, apoptotic extracellular vesicles (ApoEVs) harvested from osteogenically induced bone marrow mesenchymal stem cells (BMSCs) were further modified with the stem cell-recruiting peptide EPLQLKM (E7). By leveraging mussel-inspired adhesive peptides and bio-orthogonal click conjugation, we achieved mild but stable immobilization of the modified ApoEVs onto titanium (Ti) surfaces. The ApoEV/E7-modified surface effectively inhibited the formation of M1 macrophages and the expression of pro-inflammatory cytokines, while promoting the formation of M2 macrophages and the expression of anti-inflammatory cytokines, thereby improving the inflammatory microenvironment at the diabetic bone-implant interface. Simultaneously, the modified surface stimulated angiogenesis under diabetes-induced inflammatory conditions and enhanced osteogenic activity. *In vivo* experiments demonstrated that implants with this modified surface enhanced bone tissue regeneration and bone structure restoration at the bone-implant interface in a diabetic inflammatory environment, effectively regulating inflammation at the bone-implant interface and promoting angiogenesis. Collectively, this study suggests that the 3,4-dihydroxyphenylalanine-ApoEV/E7 biomimetic coating effectively enhances interfacial osseointegration via immunomodulation and provides a promising strategy for the design of implant coatings aimed at enhancing osseointegration under diabetic conditions.

## Highlights

• **Advanced Bio-interface Engineering:** A DOPA-anchored, click-chemistry platform stably coats Ti implants with ApoEVs.
• **Targeted Immunomodulatory Rescue:** The interface drives a pro-healing M1-to-M2 macrophage transition to resolve inflammation.
• **Enhanced Clinical Osseointegration:** This strategy restores bone-implant crosstalk to prevent early implant failure in diabetes.

## Graphical abstract

**Fabrication of the DOPA-ApoEVs/E7 system and its role in enhancing osseointegration through immunomodulated angiogenesis and osteogenesis.** A) Extraction of apoptotic extracellular vesicles (ApoEVs) from BMSCs via staurosporine (STS)-induced apoptosis. The ApoEVs are functionalized by intercalating DSPE-PEG2k-Azido (bearing a click-reactive azide group) and DSPE-PEG2k-EPLQLKM into their phospholipid bilayers. Subsequently, these modified vesicles are covalently tethered to the titanium alloy surface through a strain-promoted azide-alkyne cycloaddition reaction, reacting with DBCO-terminated, mussel-inspired biomimetic peptides pre-anchored to the substrate. B) The DOPA-ApoEVs/E7 surface facilitates macrophage mediated immune-modulation, which in turn stimulates potent angiogenic and osteogenic differentiation within inflammatory environments, ultimately achieving robust osseointegration at the bone-implant interface.

**Figure:**
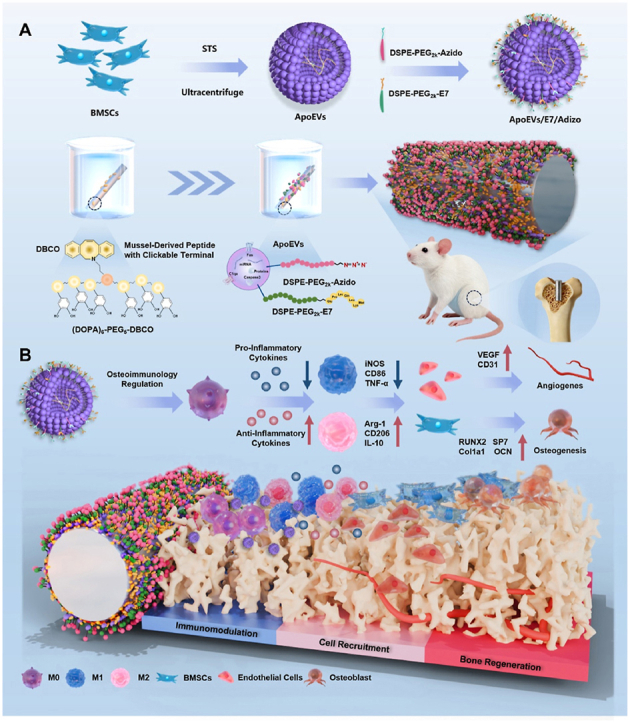

## 1 Introduction

**Scheme 1:**
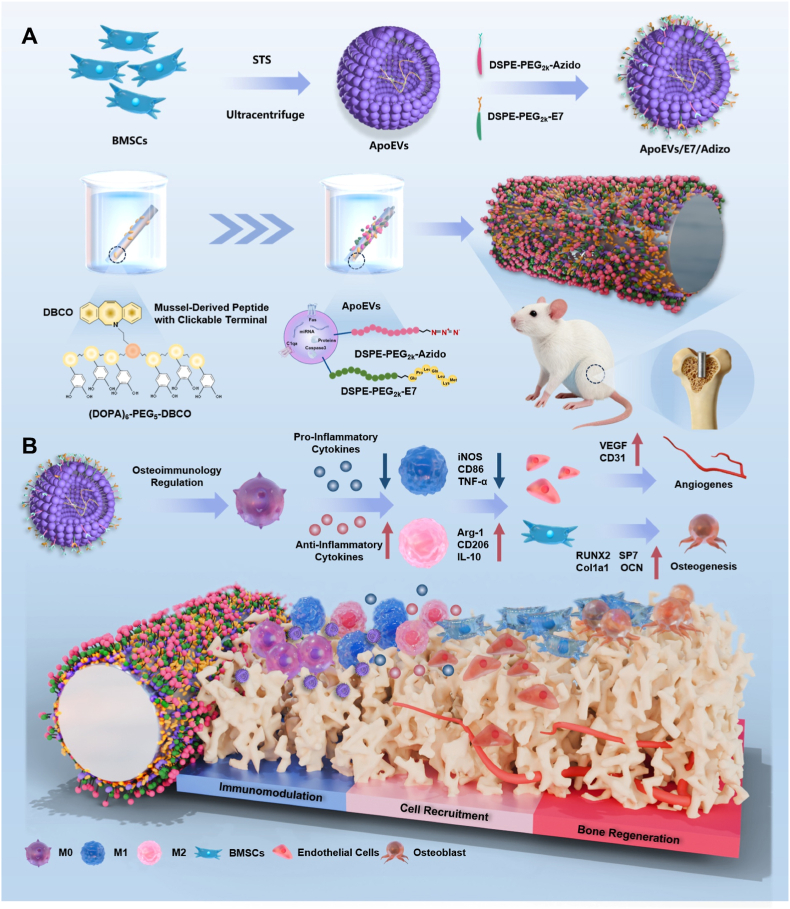
Fabrication of the 3,4-dihydroxyphenylalanine-apoptotic extracellular vesicles/EPLQLKM (DOPA-ApoEVs/E7) system and its role in enhancing osseointegration through immunomodulated angiogenesis and osteogenesis. (A) ApoEVs from bone marrow mesenchymal stem cells via staurosporine-induced apoptosis. The ApoEVs are functionalized by intercalating 1, 2-distearoyl-sn-glycero-3-phosphoethanolamine (DSPE)-polyethylene glycol (PEG)2k-Azido, bearing a click-reactive azide group and DSPE-PEG2k-E7 into their phospholipid bilayers. Subsequently, these modified vesicles are covalently tethered to the Ti alloy surface through a strain-promoted azide-alkyne cycloaddition reaction, reacting with dibenzocyclooctyne-terminated, mussel-inspired, biomimetic peptides preanchored to the substrate. (B) The DOPA-ApoEVs/E7 surface facilitates macrophage-mediated immunomodulation, which in turn stimulates potent angiogenic and osteogenic differentiation within inflammatory environments, ultimately achieving robust osseointegration at the bone-implant interface.

Implant-based therapies represent a cornerstone in the management of orthopedic and dental disorders; however, the stability of these implants is critically governed by both mechanical and environmental factors [1]. Although metallic implants are extensively used in clinical settings due to their superior mechanical strength, their inherent biological limitations and unpredictable degradation rates pose significant risks to long-term device survival [2,3]. Most metals are characterized as bioinert materials that lack the capacity for active interaction with surrounding tissues, often failing to induce a favorable phenotypic transition in immune cells [4]. The diabetic microenvironment is a systemic disorder characterized by persistent hyperglycemia, profound hypoxia, and excessive reactive oxygen species (ROS) accumulation. The interplay between hyperglycemia and hypoxia severely exacerbates oxidative stress, inducing cellular damage. Concurrently, local immune homeostasis is disrupted, trapping macrophages in the pro-inflammatory M1 phenotype and impeding their transition toward the pro-healing M2 phenotype. Consequently, this dysfunctional milieu culminates in impaired angiogenesis and compromised tissue regeneration [5,6]. Under pathological conditions such as diabetes, implant osseointegration faces substantial microenvironmental challenges of inflammatory and immune dysregulation [7]. Epidemiological studies indicate that patients with diabetes worldwide face a significantly elevated risk of severe bone loss and impaired skeletal quality. Under diabetic conditions, this accelerated bone loss dramatically increases the clinical incidence of critical-sized bone defects after trauma, which is further compounded by a suppressed regeneration capacity that leads to exceptionally high rates of healing failure. Effectively managing these persistent defects remains a major clinical challenge and a massive global healthcare burden, highlighting the urgent need for therapeutic strategies that can arrest bone loss and rescue bone regeneration[[8], [9], [10]]. The inflammatory milieu drives immune cells, predominantly macrophages, to release a cascade of pro-inflammatory cytokines; concurrently, hyperglycemia impairs mesenchymal stem cell activity by inhibiting their migration and osteogenic differentiation potential, collectively obstructing effective bone-implant integration [11,12]. Critically, bone repair and regeneration are deeply intertwined with angiogenesis; however, the pathological conditions associated with diabetes significantly impede this process. The excessive production of reactive oxygen species induced by hyperglycemia, coupled with hemorheological abnormalities and vascular endothelial dysfunction arising from chronic inflammation, severely disrupts the vascularization of bone tissue, thereby hindering osteogenic differentiation [13]. Consequently, developing implants capable of balancing the dual requirements of tissue repair and immunomodulation is essential for extending the clinical lifespan of prosthetics in the inflammatory and immunocompromised microenvironments characteristic of diabetes (see Scheme 1).

Apoptosis represents the most prevalent form of programmed cell death, occurring daily across billions of cells. During this process, dying cells secrete a substantial population of apoptotic extracellular vesicles (ApoEVs) enriched with proteins, ribonucleic acids (RNAs), deoxyribonucleic acids (DNAs), and lipids. These ApoEVs serve as critical mediators in a wide range of physiological and pathophysiological events [14,15]. Bone marrow mesenchymal stem cells (BMSCs) are multipotent stromal cells characterized by robust self-renewal capabilities, versatile multidifferentiation potential, and potent paracrine activity, serving as pivotal mediators of regenerative potential [16]. BMSCs and their cellular derivatives are recognized as potent vehicles for immunomodulation and tissue repair [17]. Angiogenesis serves as the fundamental scaffold for bone tissue restoration, as the vascular bed supplies essential nutrients and oxygen while facilitating the removal of metabolic byproducts. This vascular support is critical to sustaining cellular proliferation and collagen deposition, thereby driving the overall healing process [18]. Currently, BMSC-derived apoptotic extracellular vesicles (BMSC-ApoEVs) have been demonstrated to exert reparative effects in various immunomodulatory disorders [[19], [20], [21]]. Research indicates that ApoEVs originating from BMSCs can facilitate bone regeneration by releasing osteoinductive signals [22]. Furthermore, ApoEVs produced by organisms in response to oxygen-related environmental stress have been shown to target endothelial cells, thereby promoting angiogenesis [23]. The generation of ApoEVs is inherently safer, more straightforward, and more efficient than are metal ions or chemical pharmacological agents, offering distinct advantages in terms of biocompatibility and reduced biotoxicity. Consequently, functionalizing bone implant surfaces with differentiated BMSC-ApoEVs presents substantial potential for enhancing osseointegration by promoting vascularized bone regeneration and modulating the peri-implant immune microenvironment.

To facilitate the stable surface immobilization and functionalization of ApoEVs, the development of novel and rationally designed biomaterials for metallic surface modification is of critical importance. Marine mussels possess the ability to adhere firmly to various substrates in seawater by secreting specialized adhesive proteins, with their robust wet adhesion primarily attributed to the high concentrations of 3,4-dihydroxyphenylalanine (DOPA) within mussel foot proteins (Mfps) [24,25]. Consequently, biomimetic strategies inspired by these proteins are regarded as highly promising approaches for surface engineering. In this study, we designed a biomimetic peptide containing the catechol-functionalized amino acid DOPA, which induces strong adhesion to rigid surfaces through potent coordination interactions. The central mechanism of this approach relies on the high adhesive strength generated by repetitive catechol-functionalized amino acids. These catechol moieties facilitate seamless conjugation with diverse biomolecules and spontaneously coordinate with various metallic ions, ensuring robust, molecular-level adhesion to the implant substrate [26,27]. To leverage the full functional potential of this biomimetic peptide, we aimed to utilize the immunomodulatory activity of ApoEVs. By ensuring their stable immobilization through peptide binding, we sought to establish a synergistic angio-osteogenic coupling effect between the peptide and the ApoEVs to enhance overall tissue integration. However, as physical particles with diameters ranging from 100 to 1000 nm, methods for directly grafting ApoEVs to bioactive peptides still requires further optimization. Click chemistry offers a solution by enabling the efficient assembly of small molecular modules; specifically, the copper-free click reagent dibenzocyclooctyne (DBCO) that reacts with azide groups (-N_3_) with high specificity and efficiency while maintaining excellent biocompatibility [28]. By coupling these components via click chemistry, we ensured a versatile and highly efficient linkage that facilitates stable adhesion to the implant surface, an approach that currently demonstrates significant potential for clinical application.

In this study, we synthesized a DOPA-functionalized biomimetic peptide ([DOPA]_6_-polyethylene glycol [PEG]_5_-DBCO), incorporating a bio-orthogonal dibenzocyclooctyne (DBCO) moiety. This peptide was designed to be stably anchored onto titanium (Ti)-based substrates through robust metal-catechol coordination interactions. The terminal DBCO group was subsequently conjugated with 1, 2-distearoyl-sn-glycero-3-phosphoethanolamine (DSPE)-PEG_2k_-Azido, and this linkage facilitated the stable integration of the lipid component with the (DOPA)_6_-PEG_5_-DBCO peptide anchored on the substrate. Furthermore, to recruit BMSCs to the interface while simultaneously modulating the immune microenvironment, we synthesized a DSPE-modified E7 peptide (DSPE-PEG_2k_-E7) to functionalize the immunomodulatory BMSC-ApoEVs, ultimately constructing a novel biomimetic surface designated as DOPA-ApoEVs/E7. The regulatory effects of DOPA-ApoEVs/E7 on macrophages, as well as its capacity to modulate osteogenesis and angiogenesis within the inflammatory microenvironment of diabetes mellitus (DM), were evaluated *in vitro* and *in vivo* investigations. This study developed an innovative biomimetic coating that enhances osseointegration at the bone-implant interface by regulating vascularized bone regeneration in the inflammatory microenvironment of DM. This approach provides a new therapeutic strategy to address the clinical challenge of bone-implant interface failure under pathological immune conditions.

## 2 Results and discussion

### 2.1 Material synthesis and surface modification

**Fig. 1:**
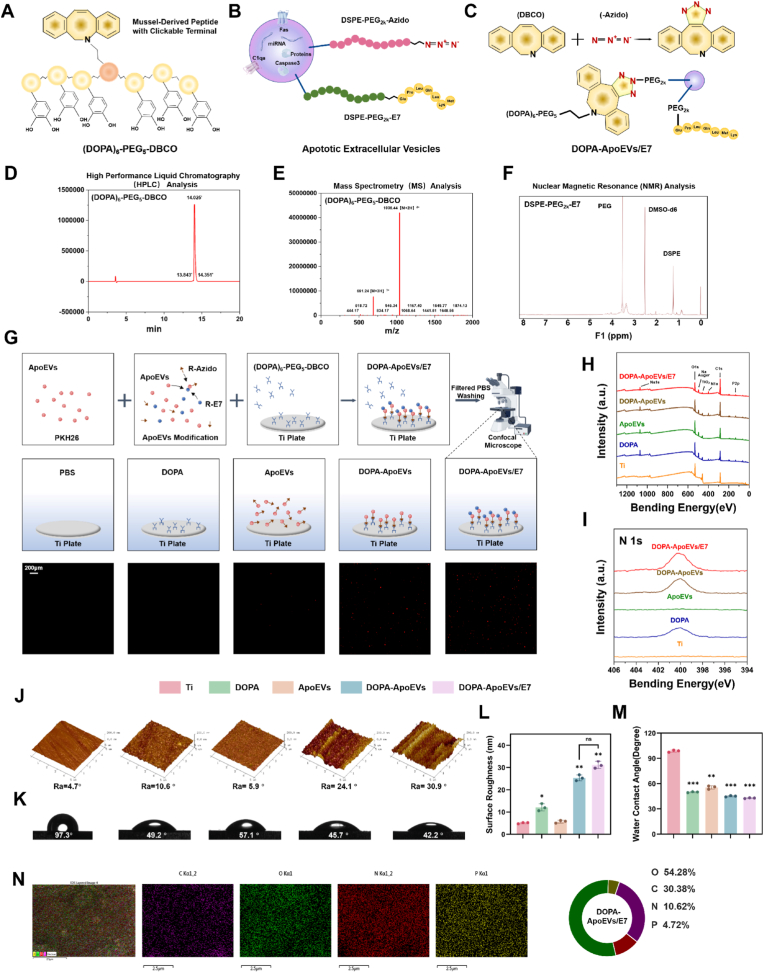
**Surface characterization of various modified materials. (**A) Schematic and structural representation of the mussel-inspired biomimetic peptide, 3,4-dihydroxyphenylalanine6-polyethyle glycol5-dibenzocyclooctyne ([DOPA]_6_-PEG_5_-DBCO), featuring a click-functionalized terminus. (B) ApoEVs modified with DSPE-PEG_2k_-Azido and DSPE-PEG_2k_-E7 peptides. (C) Bioorthogonal click reaction (Azide N_3_-DBCO cyclization). (D, E) HPLC and MS analysis of (DOPA)_6_-PEG_5_-DBCO. (F) ^l^H NMR analysis of DSPE-PEG_2k_-E7. (G) Binding of the mussel-inspired peptide to the titanium surface via catechol groups, along with fluorescence images showing PKH26-labeled ApoEVs on the modified surfaces (scale bar = 200 μm). (H, I) XPS analysis of the Ti surface and various modified surfaces. (J, L) AFM images and quantitative surface roughness analysis of the Ti surface and different modified surfaces (n = 3). (K, M) Water contact angles and quantitative analysis of the Ti surface and various modified surfaces (n = 3). (N) SEM micrographs, EDS elemental mapping, and quantitative elemental composition analysis of the DOPA-ApoEVs/E7 peptide-modified surface(n = 3). Data are expressed as the mean ± standard deviation (SD) (n = 3 per group), with statistical analysis performed using one-way ANOVA and Tukey's post-hoc test. ∗p < 0.05, ∗∗p < 0.01, and ∗∗∗p < 0.001 indicate statistical significance.

To develop a biomimetic peptide-modified surface capable of robust adhesion to titanium substrates and subsequently promoting osseointegration, we designed and fabricated a mussel-inspired peptide functionalized with a DBCO moiety. Drawing inspiration from the adhesive proteins found in mussel byssus, the synthesis was performed via a standard 9-fluorenylmethyloxycarbony (Fmoc)-based solid-phase peptide synthesis strategy (SPSS) [29]. This approach allowed for the precise incorporation of the DBCO group, providing a versatile chemical handle for further interfacial functionalization while leveraging the catechol-driven adhesive properties of the peptide to ensure stable attachment to the Ti surfaces. To ensure that a sufficient density of catechol moieties remained available for subsequent bio-orthogonal “click” conjugation, we engineered a mussel-inspired peptide, specifically (DOPA)_6_-PEG_5_-DBCO (Fig. 1A and Fig. S1A). The molecular architecture features a hexavalent DOPA motif, with each residue separated by a single amino acid spacer to prevent steric hindrance, coupled with a DBCO group via a long-chain PEG_5_ linker. This specific configuration was designed not only to leverage the robust adhesive properties of the catechol-rich sequence but also to provide the necessary spatial flexibility for the ensuing interfacial reactions through the PEGylated DBCO tether [30]. Following purification via high-performance liquid chromatography (HPLC), the mussel-inspired peptide achieved a purity exceeding 99%. Subsequent electrospray ionization mass spectrometry (ESI-MS) analysis confirmed the identity of (DOPA)_6_-PEG_5_-DBCO, yielding a [M+2H]^2+^ signal with a monoisotopic mass of 1036.44 Da. This value is in precise agreement with the theoretical molecular weight of 2070.13 Da (Fig. 1D and E). To facilitate extracellular vesicles functionalization, we used DSPE as a lipophilic anchor. Owing to its hydrophobic nature, the DSPE moiety effectively inserts into the phospholipid bilayer of the ApoEVs, providing a stable structural link for subsequent surface modifications [31]. Therefore, to ensure more efficient modification of the ApoEVs, DSPE-PEG_2k_-Azido(-N_3_) was employed as a clickable grafting ligand, facilitating the rapid conjugation of ApoEVs to the DBCO groups on the Ti surface. Additionally, we synthesized DSPE-PEG_2k_-E7 (Fig. 1C and Fig. S1B), the chemical structure of which was identified through proton nuclear magnetic resonance (^lH^ NMR) analysis (Fig. 1F). This construct was subsequently integrated with the ApoEVs in the following experimental stages.

To prepare the ApoEVs, primary BMSCs were harvested from the rat bone marrow cavity for the subsequent experimental phases. Following iterative medium changes and expansion, the BMSC population was purified to the third passage (P3). At this stage, flow cytometric analysis revealed robust expression levels of cluster of differentiation (CD) 29 (99.7%), CD44 (99.9%), and CD90 (99.9%), alongside negligible CD45 expression (0.19%) (Fig. S2A–D). This immunophenotypic profile confirmed the high purity of the isolated BMSCs, rendering them suitable for the ensuing studies [32]. Compared with those derived from standard BMSCs, ApoEVs harvested from BMSCs undergoing osteogenic differentiation exhibit superior immunomodulatory effects [33]. Consequently, following a 2-day induction period of osteogenic differentiation, we treated the BMSCs with 500 nM staurosporine (STS) to initiate apoptosis. After 12 h of exposure, terminal deoxynucleotidyl transferase dUTP nick end labeling (TUNEL) assays revealed widespread apoptotic signaling (Fig. S3A and B), a finding further corroborated by a marked decline in mitochondrial membrane potential as indicated by JC-1 staining (Fig. S3C and D). Collectively, these observations confirmed that STS treatment effectively triggered significant apoptosis in BMSCs. This was further substantiated by annexin V conjugated to fluorescein isothiocyanate, paired with propidium Iodide (Annexin V-FITC/PI) flow cytometric analysis, which clearly demonstrated a significant shift toward programmed cell death in the BMSC population (Fig. S4A and B). The above findings indicate that the ApoEVs were indeed derived from BMSCs undergoing induced apoptosis. Concurrently, the cell culture supernatant was harvested 12 h post-induction, from which ApoEVs were isolated via ultracentrifugation; the detailed isolation protocol is illustrated in Fig. S5. The physical properties of the resulting ApoEVs, including particle size distribution, zeta potential, and morphological appearance, were characterized by nanoparticle tracking analysis (NTA) using a ZetaView PMX 110 (Particle Metrix, Germany) and transmission electron microscopy (TEM) (Fig. S6A and B). The protein concentration of the isolated ApoEVs was subsequently examined by bicinchoninic acid (BCA) assay, yielding a value of 122.7 ± 6.8 ng/μL. Based on these quantitative measurements, the total yield per sample was calculated to be approximately 1.8 × 10^10^ particles, with a corresponding particle-to-protein ratio of roughly 3.2 × 10^8^ particles/μg (Fig. S7A–D). Additionally, Western blot (WB) was used to examine the expression of the characteristic apoptotic markers Fas, caspase-3, and complement C1q a-chain (C1qa). The results demonstrated a robust enrichment of these apoptotic signature proteins within the isolated ApoEVs (Fig. S6C). This successful isolation and characterization of the ApoEVs established a critical foundation for the subsequent fabrication of the bioactive coatings.

We then proceeded with the fabrication of the biomaterial coatings. Initially, the Ti rods and discs were immersed in a 0.01 mg/mL solution of (DOPA)_6_-PEG_5_-DBCO, a step designed to ensure uniform coverage of the mussel-inspired biomimetic peptide across the substrates. Concurrently, DSPE-PEG_2k_-Azido and DSPE-PEG_2k_-E7 were prepared at a 1:1 M ratio to reach a final concentration of 0.01 mg/mL, after which this mixture was integrated with the ApoEVs (Fig. 1B). Finally, the different modified surfaces were fabricated through a bio-orthogonal click chemistry reaction between the DBCO and azido moieties. Beyond the pristine titanium (Pure Ti) control, the experimental groups comprised the DOPA group, which was coated exclusively with (DOPA)_6_-PEG_5_-DBCO; the ApoEVs group, which was treated with ApoEVs alone; the DOPA-ApoEVs group, in which ApoEVs were grafted onto the (DOPA)_6_-PEG_5_-DBCO-precoated surface; and the DOPA-ApoEVs/E7 group, which included additional integration of the E7 peptide. To visually assess the robust conjugation and structural stability between the peptides and ApoEVs, we used a PKH26 fluorescent probe for tracking. Given its high lipophilicity, PKH26 stably intercalates into the lipid bilayer regions of the ApoEVs. Experimentally, the harvested ApoEVs were first co-incubated with PKH26, followed by conjugation with DSPE-PEG_2k_-Azido and DSPE-PEG_2k_-E7 under light-protected conditions. These functionalized vesicles were then utilized to fabricate the various coatings on the Ti surfaces according to the previously established protocols. Following the fabrication process, the surfaces were examined via confocal laser scanning microscopy. The resulting images revealed a markedly higher density of fluorescence-positive spots in the DOPA-ApoEVs and DOPA-ApoEVs/E7 groups than in other groups (Fig. 1G). Additionally, we evaluated the fluorescent labeling of ApoEVs at days 3 and 7 post-grafting. The experimental results indicated that the grafted ApoEVs maintained their structural integrity and stability for up to 7 days; although the vesicle count exhibited a slight decline compared to the initial state, it remained at a level comparable to the initial grafting state(Fig. S8). These observations confirm that the azide-functionalized ApoEVs successfully underwent click conjugation with the DBCO-modified DOPA layer, resulting in their stable immobilization on the Ti substrates. These findings clearly validate the efficacy of our surface modification strategy for tethering ApoEVs to Ti surfaces. Because enhanced surface roughness can promote BMSCs spreading and adhesion [34,35], we used atomic force microscopy (AFM) to evaluate the topographical alterations for different experimental groups. The images revealed a distinct increase in surface roughness in the DOPA-only group compared with the pristine Ti substrates (Fig. 1J–L). This effect was further intensified in the DOPA-ApoEVs and DOPA-ApoEVs/E7 groups, thereby confirming the successful deposition of the peptide layer and the subsequent immobilization of the extracellular vesicles. Because enhanced surface hydrophilicity can significantly promote osteoblast proliferation [36], we employed static water contact angle measurements to assess the wetting behavior of the different substrates. The analysis revealed that the peptide-modified surfaces showed a marked reduction in contact angle compared to both the pristine Ti and the ApoEVs-only groups (Fig. 1K–M). These results indicate a substantial shift toward a more hydrophilic state after functionalization. Subsequently, the elemental composition of each Ti surface was examined to verify the modification density. Within the X-ray photoelectron spectroscopy (XPS) spectra, the N 1s peak, which was primarily attributed to amide bonds within the peptide backbone, showed markedly higher intensity in the DOPA, DOPA-ApoEVs, and DOPA-ApoEVs/E7 groups than in both the control and ApoEVs-only groups. This finding provides further evidence for the successful immobilization of the peptides and ApoEVs (Fig. 1H and I). High-resolution XPS peak fitting of the N 1s spectrum clearly reveals specific —NH2 and N—O bonds characteristic of the DOPA-ApoEVs/E7 coating (Fig. S10). Complementing the XPS data, energy-dispersive X-ray spectroscopy (EDS) elemental mapping revealed that nitrogen accounted for 10.62% of the total atomic percentage, providing additional confirmation of the efficacy of the peptide-based functionalization (Fig. 1N). Furthermore, we performed SEM-EDS mapping to analyze the surface elemental distribution of the grafted Ti rods 4 weeks post-operation (Fig. S9). The elemental ratios at week 4 were highly consistent with those of the newly grafted surfaces, definitively confirming the long-term structural and chemical stability of our ApoEVs immobilization strategy. BMSCs-ApoEVs exhibit excellent biocompatibility, and their preparation and storage are notably straightforward and efficient. Given their diverse cargo of proteins, RNA, and DNA, ApoEVs possess multifaceted biological functionality and are increasingly recognized as potent bioregulatory agents [19,23,31]. In contrast, contemporary surface modification techniques for biomaterials remain limited. For instance, plasma-sprayed coatings are frequently prone to brittle fracture, whereas coatings produced via electrochemical deposition often show poor interfacial adhesion to metallic substrates. Although micro-arc oxidation can generate oxide films, the resulting surfaces are often excessively rough, and the fatigue strength of the material may be compromised. Ultimately, these limitations have hindered the full realization of the desired biological functions in current modification strategies [[37], [38], [39]]. Maintaining the intrinsic bioactivity of therapeutic components remains a paramount yet challenging prerequisite for successfully constructing bioinspired implant coatings. First, while strict sterility is non-negotiable for translational implants, harsh sterilization techniques (such as autoclaving or heavy irradiation) inevitably denature fragile biologics; thus, the functional ApoEVs were prepared under strictly aseptic conditions. Concurrently, all synthetic biocomponents underwent rigorous sterilization prior to functionalization. Furthermore, the immobilization of each component was achieved via a highly efficient and mild bioorthogonal click chemistry network. This conjugation reaction proceeds rapidly under physiological, ambient conditions, successfully circumventing the risks of protein denaturation or vesicular disruption, thereby ensuring that the components exert potent immunomodulatory and therapeutic efficacies *in vivo.* Currently, the prevailing design paradigm for biomaterial coatings centers on the precise coupling of biological substrates with specific functional units. This strategy typically involves using biocompatible materials as scaffolds, doped with low concentrations of functional elements such as Cu^2+^, Mg^2+^, Zn^2+^ or macromolecular agents, such as bone morphogenetic protein −2. However, the complex physiological microenvironment *in vivo* often leads to uncontrolled ion release rates or catastrophic coating delamination. Furthermore, many bioactive molecules exhibit poor intrinsic stability, making it difficult to satisfy the clinical demand for implants that remain robust and reliable over the long term [40,41]. At present, synthetic peptides are increasingly favored for biomaterial surface modifications because they offer sustained bioactivity, enhanced biosafety, and structural robustness compared with traditional agents. Their inherent chemical versatility also enables precise modifications, creating significant opportunities for the development of customized biological functions [42]. In summary, the mussel-inspired adhesive peptide engineered in this study was successfully conjugated with ApoEVs via bio-rthogonal click chemistry. This approach yielded an effectively functionalized bioactive surface and presents a novel strategy for advanced biomaterial surface modification.

### 2.2 Biocompatibility evaluation

**Fig. 2:**
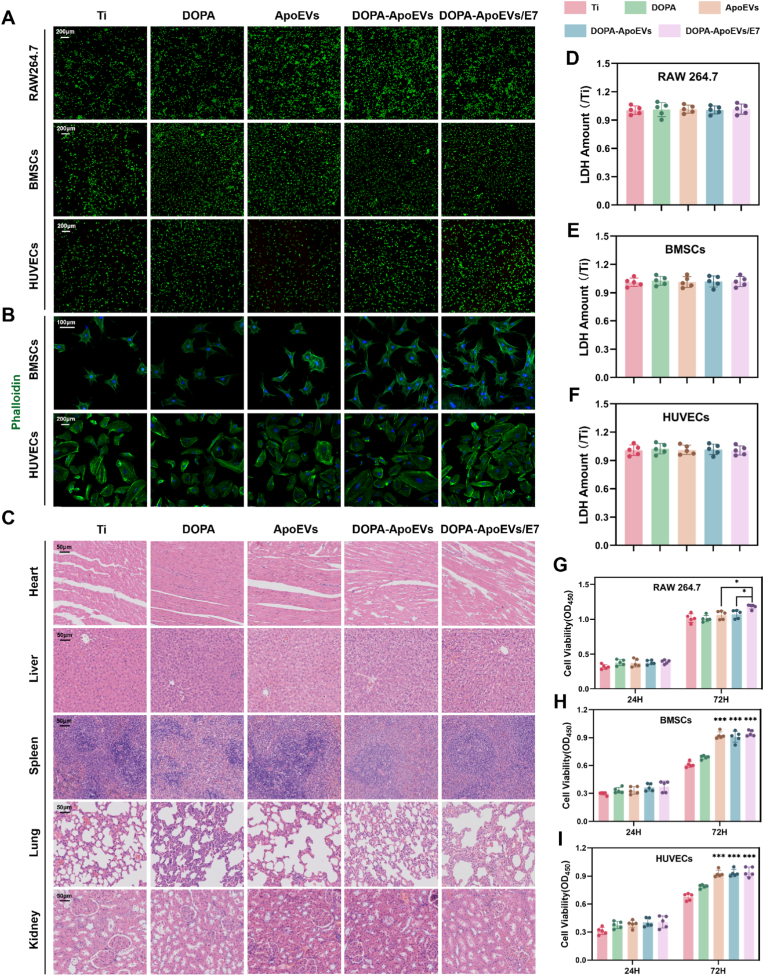
***In vitro* and *in vivo* biocompatibility assessment of various modified surfaces.** (A) Live/dead staining of RAW 264.7 cells, BMSCs, and HUVECs cultured on different modified surfaces(scale bar = 200 μm). (B) Cytoskeletal staining of BMSCs(scale bar = 100 μm) and HUVECs(scale bar = 200 μm) on various modified surfaces using FITC-labeled phalloidin. (C) H&E staining of the heart, liver, spleen, lungs, and kidneys from SD rats 8 weeks after implantation with titanium rods featuring different modified surfaces. (D–F) LDH cytotoxicity assays of RAW 264.7 cells, BMSCs, and HUVECs cultured on different modified surfaces ((scale bar = 50 μm)n = 5). (G–I) CCK-8 assays evaluating the viability of RAW 264.7 cells, BMSCs, and HUVECs cultured on various modified surfaces at 24 h and 72 h (n = 5). Data are expressed as the mean ± standard deviation (SD), with statistical analysis performed using one-way ANOVA and Tukey's post-hoc test. ∗∗∗p < 0.001 indicates statistical significance.

In this study, RAW 264.7, BMSC and human umbilical vein endothelial (HUVEC) cells were used to evaluate the biocompatibility of the various surfaces. We initially cultured the three cell types under standard conditions and then replaced the medium with a conditioned medium (CM) for DM-CM, which contained 25 mM high glucose and 100 ng/mL lipopolysaccharide to simulate the DM microenvironment. We then performed live/dead staining using calcein-acetoxymethyl ester/propodium iodide (calcein-AM/PI) to assess the viability of these three cell types across the different groups. The results demonstrated that only a small number of red fluorescence-positive dead cells were visible in any group, indicating no significant difference in the live-to-dead cell ratios across the experimental groups (Fig. 2A). To further characterize the potential cytotoxicity of the modified surfaces, we quantified the secretion of lactate dehydrogenase (LDH) from the seeded cells. Following 24 h of co-incubation, LDH levels remained consistently low with no significant differences observed among groups. These results indicate that the peptide-modified surfaces exert no discernible toxic effects on the cells (Fig. 2D–F). E7 is a bioactive peptide known for its ability to facilitate stem cell recruitment. To evaluate the *in vitro* recruitment efficacy of E7, we performed phalloidin staining of the cytoskeleton to assess the attachment and spreading of BMSCs. Confocal laser scanning microscopy images revealed increased filamentous actin (F-actin) expression in BMSCs on the modified titanium surfaces compared with the pristine Ti group. Notably, BMSCs in the DOPA-ApoEVs/E7 group exhibited more pronounced elongation and proliferative activity, suggesting that the E7-functionalized surfaces effectively recruited a higher density of BMSCs and enhanced their proliferation (Fig. 2B). In addition to the stem cell response, we characterized the attachment and spreading of HUVECs; however, no discernible differences were observed across the various experimental groups (Fig. 2B). To further ascertain the influence of these modified surfaces on cellular proliferation, CCK-8 assays were conducted using RAW 264.7 cells, BMSCs, and HUVECs at 24 h and 72 h post-seeding. Absorbance was recorded at 450 nm using a microplate reader. The resulting data indicated that although RAW 264.7 cells exhibited no significant differences in optical density after 72 h, significantly higher absorbance was observed for both BMSCs and HUVECs in the ApoEVs, DOPA-ApoEVs, and DOPA-ApoEVs/E7 groups (Fig. 2G–I). These findings suggest that the functionalized surfaces effectively enhanced the proliferative capacity of both BMSCs and HUVECs. Studies have indicated that fatty vacuoles may be observed in the livers of diabetic model rats, likely because of dysfunctional lipid metabolism in hepatocytes under hyperglycemic conditions; however, this phenomenon is not consistently observed across all diabetic models [[43], [44], [45]]. To eliminate this confounding factor, we utilized healthy Sprague-Dawley (SD) rats to evaluate whether implants with various surface modifications exhibit systemic organ toxicity. To extend these findings, we further evaluated the biocompatibility of the peptide-modified Ti surfaces *in vivo*. The surface-engineered Ti rods were surgically implanted into the femurs of SD rats. Following a 2-month implantation period, hematoxylin and eosin (H&E) staining was performed for histopathological analysis of the major organs, which confirmed the absence of systemic organ toxicity across all modification groups (Fig. 2C). Collectively, these validation results demonstrate that our biomimetic peptide-modified coating significantly strengthened cellular adhesion and facilitated the robust growth of Raw 264.7 cells, BMSCs, and HUVECs without eliciting discernible biological toxicity. These outcomes underscore the superior biocompatibility of the modified surfaces and highlight their significant potential for enhancing osteogenesis.

### 2.3 Evaluation of osteoimmunomodulatory effects on modified surfaces

**Fig. 3:**
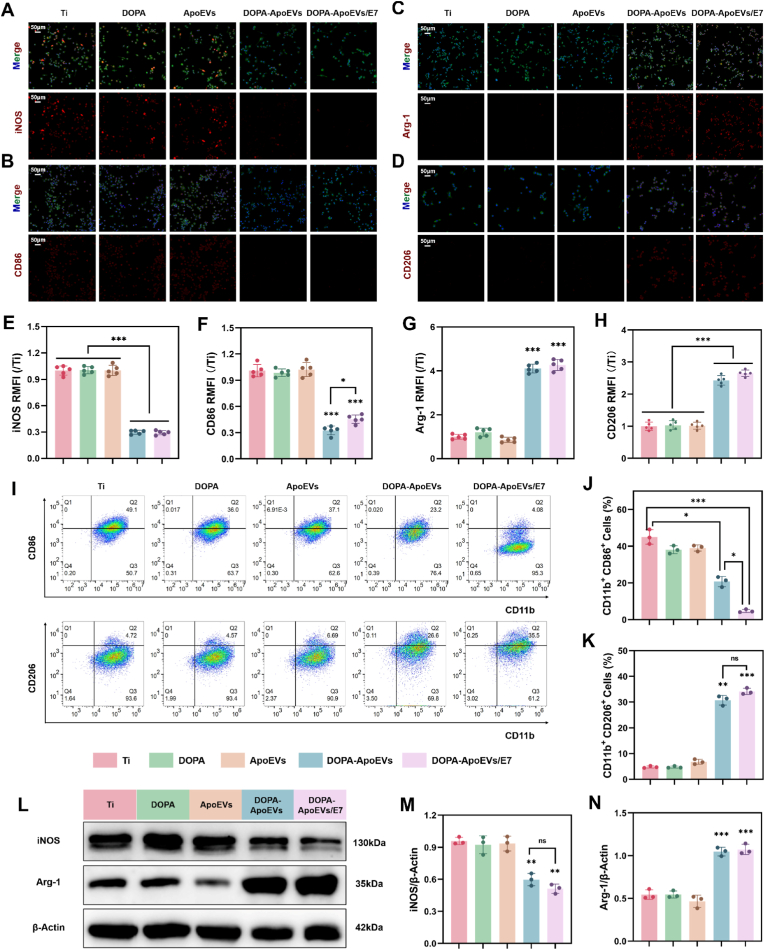
***In vitro* immunomodulatory effects of different modified surfaces on macrophages. (**A–D) Representative immunofluorescence images of RAW 264.7 cells seeded on titanium discs with various modifications and cultured in diabetes mellitus (DM)-mimicking medium. Green fluorescence represents the cytoskeleton; red indicates M1 markers (iNOS and CD86) and M2 markers (Arg-1 and CD206); blue DAPI staining represents nuclei (scale bar = 50 μm). (E–H) Quantitative analysis of the fluorescence intensity for the respective marker proteins (n = 5). (I–K) Flow cytometric analysis showing the expression of M1 marker CD86 and M2 marker CD206 on different surfaces, with corresponding quantitative results (n = 3). (L–N) Western blot (WB) analysis of iNOS and Arg-1 protein expression levels alongside their respective quantitative assessments (n = 3). Data are expressed as the mean ± standard deviation (SD), with statistical analysis performed using one-way ANOVA and Tukey's post-hoc test. ∗∗p < 0.01 and ∗∗∗p < 0.001 indicate statistical significance and ns indicate no statistical significance.

In a DM environment, dysregulated macrophage polarization phenotypes disrupt the essential cellular crosstalk between the implant and the surrounding bone tissue. Under the inflammatory conditions associated with DM, this impaired immunomodulatory mechanism leads to exacerbated fibrous capsule formation, which directly compromises the functional integration and long-term biological stability of the implant [46]. Upon implantation, exogenous materials tend to exacerbate the peri-implant immune response; specifically, massive recruitment of pro-inflammatory M1 macrophages during the initial phase frequently culminates in deficient osseointegration at the bone-implant interface. This cascade often triggers early clinical failure and prosthetic loosening [47]. In a DM inflammatory microenvironment, the resolution of pro-inflammatory M1 macrophage activation and the subsequent transition toward anti-inflammatory M2 macrophage polarization represent fundamental mechanisms underlying implant osteoimmunomodulation. In the post-implantation phase, the transition from early acute inflammation to sustained chronic inflammation, coupled with alterations in the immune microenvironment, disrupts the secretory balance between pro- and anti-inflammatory cytokines. This shift further dictates the polarization trajectory of macrophages, ultimately impeding peri-implant osseointegration and culminating in clinical failure [28]. Consequently, in the context of a diabetic inflammatory milieu, suppressing M1 polarization and the concomitant secretion of pro-inflammatory factors, while simultaneously upregulating M2 polarization and anti-inflammatory signaling, emerges as a pivotal strategy for enhancing the efficacy of implant osseointegration. To thoroughly validate the efficacy and rationale of our *in vitro* diabetic model, we initially prepared a series of simulated diabetic microenvironment media featuring distinct combinational concentrations of high glucose (25 mM and 30 mM) and LPS (10 ng/mL and 100 ng/mL). We then systematically evaluated the corresponding phenotypic alterations of macrophages, BMSCs, and HUVECs under these varied culture conditions. Our findings revealed that the combination of 25 mM glucose and 100 ng/mL LPS represented the optimal and most robust condition to simultaneously trigger pro-inflammatory M1 macrophage polarization(Fig. S11A and B), suppress BMSC osteogenic differentiation(Fig. S11C–E), and impair HUVEC angiogenesis (Fig. S11F–I). This multidimensional verification firmly substantiates the validity and appropriateness of our established pathological model. Subsequently, RAW 264.7 cells were seeded onto the various functionalized substrates and maintained in this DM-mimicking medium. Immunofluorescence staining was then performed on the RAW 264.7 cells to evaluate their polarization trajectories when cultured on the different surfaces. As illustrated by the immunofluorescence imaging and corresponding quantitative analysis in Fig. 3A–H, the surfaces of the DOPA-ApoEVs and DOPA-ApoEVs/E7 groups exhibited a substantial increase in the abundance of the M2 macrophage markers arginase-1 (Arg-1) and CD206. Concurrently, the abundance of the pro-inflammatory M1 markers inducible nitric oxide synthase (iNOS) and CD86 was markedly suppressed. In contrast, a reciprocal trend was observed in the Ti, DOPA, and ApoEVs-only groups. Collectively, these observations suggest that surface functionalization with DOPA-tethered ApoEVs creates a potent modulatory interface that effectively directs macrophage activation toward a pro-healing M2 phenotype. Subsequently, we quantified the macrophage phenotypes using flow cytometry and WB analysis. The results indicated a significantly higher proportion of CD11b^+^CD86^+^ cells (M1 macrophages) within the Ti, DOPA, and ApoEVs cohorts (Fig. 3I and J), which correlated with elevated expression levels of iNOS protein (Fig. 3L and M). In contrast, the DOPA-ApoEVs and DOPA-ApoEVs/E7 groups showed a markedly elevated percentage of CD11b^+^CD206^+^ cells (M2 macrophages) relative to the other three groups (Fig. 3I and K). This phenotypic shift was further corroborated by the corresponding increase in Arg-1 protein expression (Fig. 3L and N). These findings collectively suggest that the functionalized surface, following the conjugation of DOPA and ApoEVs, effectively increases the M2/M1 macrophage ratio. Interestingly, while we engineered an additional variant by grafting the E7 peptide onto the modified surface, the data indicate that ApoEVs remain the primary driver of macrophage phenotypic polarization. However, those ApoEVs not tethered to DOPA via bioorthogonal click chemistry were incapable of independently modulating macrophage polarization, which may be intrinsically linked to the inherent antioxidant properties of DOPA [48]. To more discernibly elucidate whether our engineered coating could specifically reverse the biological dysregulations induced by the diabetic microenvironment, a normal medium control group was established to benchmark macrophage polarization and the osteogenic differentiation of BMSCs *in vitro*. Literature indicates that under physiological conditions, macrophages reside in an uncommitted M0 state, characterized by baseline expressions of both M1 and M2 markers. Although RAW 264.7 cells are notoriously sensitive and prone to “unintended polarization” toward the pro-inflammatory M1 phenotype due to minor environmental fluctuations or routine handling, our observations confirmed that neither M1 nor M2 markers exhibited prominent expression in the non-diabetic control group (Fig. S12). Furthermore, osteogenic differentiation was unimpeded in the normal medium cohort, as evidenced by the robust expression of ALP at day 7 and ARS at day 21 across all groups (Fig. S13). Parallel comparisons with the diabetic experimental cohorts clearly demonstrate that DOPA-ApoEVs and DOPA-ApoEVs/E7 possess the specific capacity to rescue diabetic microenvironment-induced pathological perturbations, effectively resolving diabetic inflammation while driving osteogenic commitment. To further delineate the cytokine expression profiles across the various modified surfaces within a DM-mimicking milieu, we conducted an analysis of the transcriptional expression. Quantitative reverse transcription polymerase chain reaction (RT-qPCR) results revealed a robust upregulation of pro-inflammatory markers, tumor necrosis factor alpha (TNF-α) and interleukin-1 beta (IL-1β), in the Ti, DOPA, and ApoEVs cohorts. Conversely, the DOPA-ApoEVs and DOPA-ApoEVs/E7 groups favored an anti-inflammatory signature, characterized by significantly elevated expression of interleukin 6 (IL-6) and interleukin 10 (IL-10) (Fig. S14A–D). Beyond these immunomodulatory shifts, we observed that the pro-angiogenic gene vascular endothelial growth factor (VEGF) was also upregulated in the DOPA-ApoEVs and DOPA-ApoEVs/E7 groups (Fig. S14E), suggesting a potential synergy between immune regulation and vascularization. One of the central pathological hallmarks of the diabetic microenvironment is hyperglycemia-induced excessive generation of reactive oxygen species (ROS), which severely impairs both angiogenesis and osteogenic differentiation. To address this issue, we performed cellular ROS fluorescence staining (utilizing the DCFH-DA probe) on macrophages exposed to a simulated diabetic inflammatory milieu. As anticipated, the experimental data revealed that all DOPA-containing formulations significantly attenuated intracellular ROS fluorescence intensity compared to the pristine Ti control, directly validating the potent antioxidant and ROS-scavenging capabilities of DOPA. Crucially, DOPA-ApoEVs and DOPA-ApoEVs/E7 exhibited an even superior efficiency in clearing ROS (Fig. S15). This further enhanced therapeutic efficacy is likely attributed to the prominent immunomodulatory capacity of ApoEVs within the diabetic inflammatory microenvironment, which works synergistically with DOPA to resolve oxidative stress.To complement the transcriptional data, cytokine secretion was quantified via ELISA. The results demonstrated that TNF-α levels in the Ti, DOPA, and ApoEVs cohorts were markedly elevated compared with those in the DOPA-ApoEVs and DOPA-ApoEVs/E7 groups. Conversely, IL-10 secretion was substantially elevated in the DOPA-ApoEVs and DOPA-ApoEVs/E7 functionalized groups (Fig. S16A and B). Following a similar trend, manifestation of VEGF was substantially enhanced in both the DOPA-ApoEVs and DOPA-ApoEVs/E7 groups (Fig. S16C). To evaluate whether the standalone E7 peptide participates in immunomodulation within a diabetic inflammatory environment, we established an independent E7 group to monitor the alterations in macrophage polarization phenotypes. The results demonstrated that the standalone E7 peptide exerted no discernible effect on modulating macrophage polarization under diabetic inflammatory conditions (Fig. S17). Collectively, these findings demonstrate that surface modification via DOPA-ApoEVs effectively attenuates the elevated release of pro-inflammatory cytokines typical of the DM milieu, while simultaneously enhancing the secretion of anti-inflammatory factors, thereby improving the overall diabetic immune microenvironment. Beyond this immunomodulatory effect, the observed upregulation of VEGF suggests that the synergistic modification of DOPA and ApoEVs facilitates a proangiogenic environment even under diabetic conditions. This capacity to create favorable conditions for vascularization likely represents a critical determinant of enhancing the subsequent osseointegration of the implant.

### 2.4 Analysis of immunomodulatory mechanisms

**Fig. 4:**
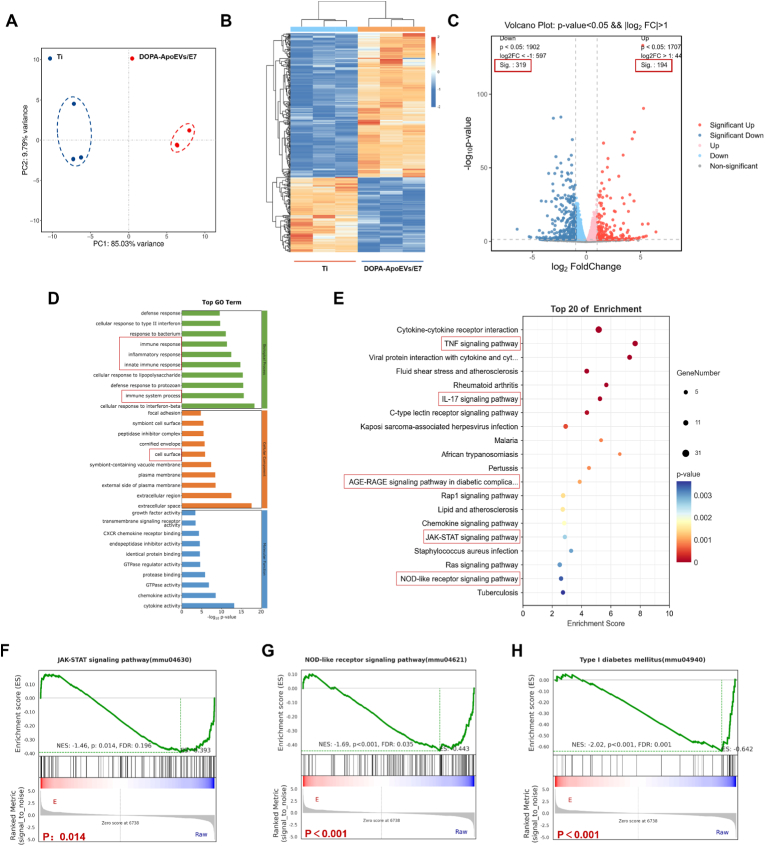
**Transcriptomic sequencing reveals the immunomodulatory mechanisms of DOPA-ApoEVs/E7-modified surfaces under DM conditions.** (A) Principal component analysis (PCA) of differentially expressed genes (DEGs) in the Ti and DOPA-ApoEVs/EPLQLKM groups. (B) Heatmap showing the expression profiles of DEGs. (C) Volcano plot representing the distribution of DEGs. (D) GO enrichment analysis of the DEGs. (E) Top 20 enriched pathways identified via the Kyoto Encyclopedia of Genes and Genomes (KEGG) database. (F–J) Gene Set Enrichment Analysis (GSEA) highlighting signaling pathways associated with inflammatory responses.

To explore the immunomodulatory mechanisms governing the biomimetic peptide-modified surfaces, we performed RNA-seq transcriptomic analysis on RAW 264.7 cells cultured on pristine Ti and DOPA-ApoEVs/E7 substrates. Principal component analysis (PCA) (Fig. 4A), alongside correlation heatmaps, gene expression boxplots, regional distribution plots, and clustering analysis, collectively indicated that the sequencing data met all necessary quality standards (Fig. S18A–D). These metrics validated the reliability of the RNA-seq results for subsequent interpretation. Volcano plot analysis revealed that the DOPA-ApoEVs/E7-modified group, relative to the pristine Ti group, exhibited 194 upregulated and 319 downregulated genes (Fig. 4B). Furthermore, the heatmap clearly delineated the differentially expressed genes (DEGs) among the different cohorts (Fig. 4C), suggesting that transcriptomic reprogramming was strongly influenced by the DOPA-ApoEVs/E7 surface modification. Subsequent Gene Ontology (GO) enrichment analysis of these DEGs indicated that, compared with the control group, the biological processes altered by DOPA-ApoEVs/E7 were primarily concentrated in inflammatory response and immune regulation (Fig. 4D). Additionally, Kyoto Encyclopedia of Genes and Genomes (KEGG) pathway enrichment analysis was employed to pinpoint the core immunomodulatory signaling pathways driving these functional shifts (Fig. 4E). The results indicated a significant downregulation of the JAK-STAT, NF-κB and NOD-like receptor (NLR) signaling pathways in the DOPA-ApoEVs/E7-modified group (Fig. 4F–H). These specific pathways are known to exert substantial effects on the modulation of immune responses and the progression of inflammatory activity. Structurally, ApoEVs are equipped with a diverse repertoire of functional proteins and miRNAs. In addition to modulating immune signal transduction, the DOPA-ApoEVs/E7-modified surface may also influence broader cellular activities and molecular functions, particularly cell adhesion and CXC chemokine receptor binding. The Western blot verification corroborated these transcriptomic insights at the translational level, demonstrating that both the DOPA-ApoEVs and DOPA-ApoEVs/E7 coatings significantly downregulated the protein expression of key regulators within the JAK-STAT and NF-κB signaling pathways. This targeted suppression directly addresses the persistent hyperactivation of JAK-STAT and NF-κB under diabetic inflammatory conditions, which typically drives chronic M1 macrophage polarization. Furthermore, the marked downregulation of the pivotal pro-inflammatory chemokine CXCL1 confirmed that the DOPA-ApoEVs and DOPA-ApoEVs/E7 coatings effectively inhibited the excessive infiltration of inflammatory cells within the diabetic microenvironment(Fig. S19). Taken together, these findings demonstrate that the DOPA-ApoEVs and DOPA-ApoEVs/E7 coatings successfully reverse the hostile diabetic inflammatory niche via the concurrent blockade of the JAK-STAT/NF-κB axes and CXCL1-mediated signaling, thereby establishing a highly favorable environment for subsequent angiogenesis and osteogenic differentiation. Collectively, these processes play an instrumental role in orchestrating the peri-implant immunomodulatory landscape and the subsequent stages of osseointegration.

### 2.5 Promotion of *in vitro* angiogenesis via immunomodulation

**Fig. 5:**
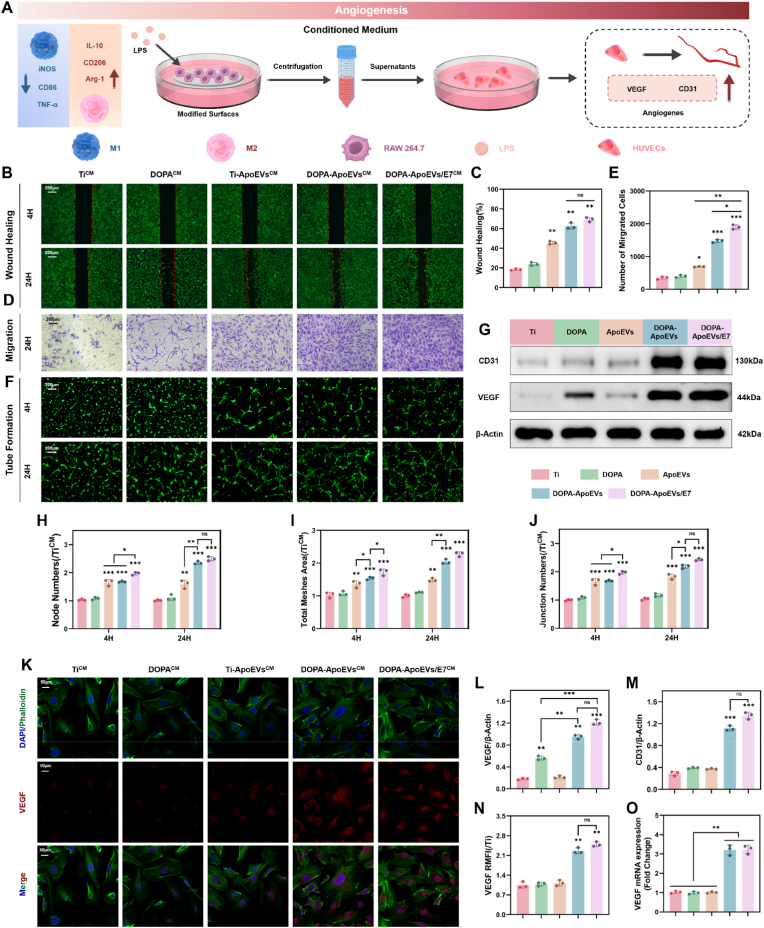
**DOPA-ApoEVs-modified surfaces promote angiogenesis under DM inflammatory conditions.** (A) Schematic illustration of the experimental design. (B, C) Wound healing assay of HUVECs cultured in conditioned media and the corresponding quantitative analysis (scale bar = 200 μm, n = 3). (D, E) Transwell migration assay of HUVECs and quantitative results (scale bar = 200 μm, n = 3). (F, H-J) Tube formation assay at 4 h and 24 h with associated quantitative analysis (scale bar = 200 μm, n = 3). (G, L–M) Western blot analysis and quantification of VEGF and CD31 expression levels (n = 3). (K, N) Immuno-fluorescence staining for VEGF expression and the corresponding quantitative results (scale bar = 50 μm, n = 3). O) RT-qPCR analysis of the angiogenic marker VEGF at the mRNA level (n = 3). Data are expressed as the mean ± standard deviation (SD), with statistical analysis performed using one-way ANOVA and Tukey's post-hoc test. ∗p < 0.05, ∗∗p < 0.01, and ∗∗∗p < 0.001 indicate statistical significance and ns indicate no statistical significance.

Angiogenesis is a critical determinant of successful osseointegration at the bone-implant interface, occurring throughout the early stages of bone repair and serving as a vital conduit for the transport of nutrients and osteogenic cells [49]. Building on our previous findings that the modified surfaces upregulated VEGF gene expression and facilitated VEGF release under DM conditions, we investigated whether these functionalized substrates could promote angiogenesis through immunomodulatory pathways. To this end, we first collected macrophage-derived conditioned media, which were subsequently supplemented with 25 mM glucose and 100 ng/mL of LPS. This formulation yielded a conditioned medium (CM) that effectively mimicked the DM microenvironment, providing a standardized platform for evaluating the angiogenic impact of the various modified coatings (Fig. 5A). First, to assess HUVEC migratory capacity, a scratch assay was performed to determine cell motility. Compared with the Ti^CM^, DOPA^CM^, and ApoEVs^CM^ cohorts, the wound closure rates in the immunomodulatory DOPA-ApoEVs^CM^ and DOPA-ApoEVs/E7^CM^ groups were significantly more pronounced after 24 h (Fig. 5B and C). Furthermore, the Transwell migration assay corroborated these findings, demonstrating a significant increase in cell migration within the DOPA-ApoEVs^CM^ and DOPA-ApoEVs/E7^CM^ groups following a 24 h incubation period (Fig. 5D and E). Taken together, these findings confirm that the CM harvested from the various surfaces effectively stimulates HUVEC migration. After incubation in the respective CM for 4 h and 24 h, we quantified the number of nodes and junctions, as well as the total mesh area across all groups. Compared with the Ti cohort, the ApoEVs group showed higher values for these angiogenic parameters; however, these enhancements were statistically more pronounced in the DOPA-ApoEVs ^CM^ and DOPA-ApoEVs/E7^CM^ groups (Fig. 5F–H–J). These observations suggest that although ApoEVs alone can stimulate angiogenesis under inflammatory conditions, dual modification with DOPA and ApoEVs further augments angiogenic capacity in a DM environment by actively modulating the immune microenvironment. To further validate these observations at the molecular level, we conducted RT-qPCR and WB analyses to elucidate the expression of pivotal angiogenic markers. The findings revealed that both the DOPA-ApoEVs ^CM^ and DOPA-ApoEVs/E7 ^CM^ cohorts exhibited a substantial upregulation of VEGF mRNA levels (Fig. 5O). Consistent with the genomic data, we performed a quantitative analysis of protein levels for VEGF and CD31 protein levels. Our results revealed a significant elevation in the protein expression of both VEGF and CD31 across these groups (Fig. 5G and L-M). Moreover, immunofluorescence staining for VEGF and CD31 yielded complementary results (Fig. 5K and N, and Fig. S20A and B), further corroborating the potent proangiogenic capacity of the modified surfaces. In the inflammatory milieu of DM, reconstruction of the osteoimmune environment can foster a favorable niche for neovascularization, thereby accelerating the osseointegration of implants [50]. Collectively, these findings substantiate that the synergistic modification of surfaces with DOPA and ApoEVs facilitates enhanced angiogenesis within a diabetic milieu via immunomodulatory pathways. By fostering a pro-angiogenic environment even under chronic inflammatory conditions, these functionalized surfaces provide a robust foundation for achieving efficient osseointegration at the bone-implant interface.

### 2.6 Promotion of *in vitro* osteogenesis via immunomodulation

**Fig. 6:**
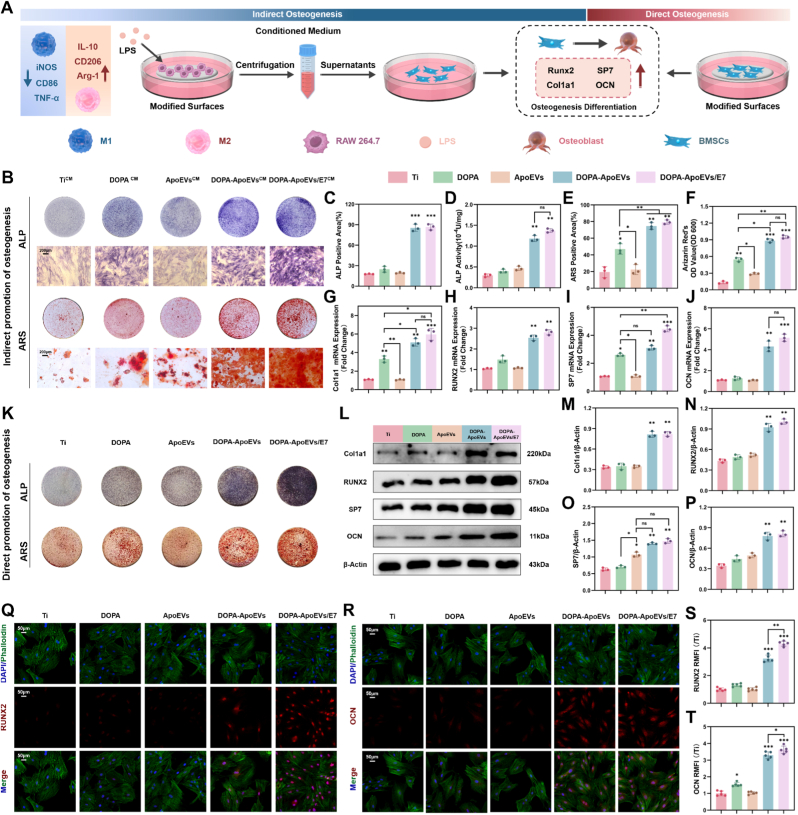
**DOPA-ApoEVs/E7-modified surfaces promote direct and indirect osteogenesis under DM inflammatory conditions.** (A) Schematic illustration of the experimental design. (B–F) Bone marrow-derived mesenchymal stem cells (BMSCs) cultured in conditioned medium supplemented with osteogenic induction factors; Compared with the Ti group, ALP and ARS staining were performed to evaluate osteogenesis, followed by corresponding quantitative analysis, (scale bar = 200 μm, n = 3). (G–H) RT-qPCR assessment of osteogenesis-related genes (Col1a1, RUNX2, Sp7, and OCN) in BMSCs cultured in osteogenic CM compared with the Ti group (n = 3). (K) BMSCs seeded directly onto Ti discs with different modified surfaces in osteogenic CM; Compared to the Ti group, ALP and ARS assays were utilized to evaluate the osteogenic effect. (L–P) Western blot analysis of osteogenic markers along with the quantitative results (n = 3). (Q–T) Immunofluorescence staining and quantitative analysis of osteogenic hallmark proteins RUNX2 and OCN compared with the Ti group in BMSCs; green: cytoskeleton, red: osteogenic markers (RUNX2 or OCN), blue: nuclei (scale bar = 200 μm, n = 5). Data are expressed as the mean ± standard deviation (SD), with statistical analysis performed using one-way ANOVA and Tukey's post-hoc test. ∗p < 0.05, ∗∗p < 0.01, and ∗∗∗p < 0.001 indicate statistical significance and ns indicate no significance.

Osteoblastic differentiation is highly sensitive to the surrounding microenvironment. The chronic inflammatory milieu induced by DM causes systemic dysregulation of cytokine release, which likely represents a major barrier to osteoblast maturation and subsequent bone repair [[51], [52], [53]]. To determine whether our functionalized surfaces could counteract these effects and enhance osteogenic function through immunomodulation, we supplemented the previously described DM-mimicking conditioned media (CM) with osteogenic induction factors. Accumulating evidence indicates that the diabetic inflammatory environment significantly impairs BMSCs viability and suppresses osteoblast lineage commitment [54]. Consequently, we systematically evaluated the effects of the various surface modifications on both direct and indirect osteogenesis (Fig. 6A). Initially, we assessed the indirect osteogenic capacity of the functionalized surfaces. Following a 2-day expansion period, the culture medium for primary BMSCs was replaced with the previously formulated DM-mimicking CM, supplemented with osteogenic induction factors. Following 7 days, alkaline phosphatase (ALP) staining was performed, and Alizarin Red S (ARS) staining was performed on day 21 to evaluate early-stage differentiation and long-term mineralized matrix formation, respectively. Under diabetic conditions, the ALP-positive area and corresponding enzymatic activity in the DOPA-ApoEVs^CM^ group were significantly higher than those in the Ti^CM^, DOPA^CM^, and ApoEVs^CM^ cohorts. This enhancement was even more pronounced in the DOPA-ApoEVs/E7^CM^ group (Fig. 6B–D). Consistent with this, ARS staining demonstrated that the immunomodulatory DOPA-ApoEVs^CM^ and DOPA-ApoEVs/E7^CM^ groups exhibited greater calcium nodule deposition than other cohorts, with the most prominent increase observed in the DOPA-ApoEVs/E7^CM^ group (Fig. 6B–E–F). This enhanced osteogenic outcome likely stems from the recruitment capacity of the E7 peptide, which acts as a chemoattractant for BMSCs and works in synergy with the immunomodulatory potency of ApoEVs to enhance indirect osteogenesis within the diabetic inflammatory microenvironment. To further investigate the specific effects of E7 and ApoEVs on osteogenic differentiation, we evaluated the osteogenic performance of the standalone E7 group alongside the Ti and DOPA-ApoEVs/E7 groups. The experimental results demonstrated that compared with the Ti group, the E7 peptide group exhibited increases in the ALP-positive area and ALP activity at day 7, as well as enhanced ARS staining at day 21 (Figure S21 A–E). However, these levels were significantly lower than those observed in the DOPA-ApoEVs/E7 group. To substantiate these findings at the molecular level, RT-qPCR and WB analyses were conducted to quantify the expression of key osteogenic markers. The results confirmed that both the DOPA-ApoEVs^CM^ and DOPA-ApoEVs/E7^CM^ groups demonstrated significant upregulation of the gene and protein expression levels of collagen type I alpha 1 (Col1a1), runt-related transcription factor 2 (RUNX2), osterix (SP7), and osteocalcin (OCN) (Fig. 6G–J, L–P). Immunofluorescence staining was used to assess the protein expression of OCN after 3 days and RUNX2 after 10 days of osteogenic induction. The findings indicated that, relative to the other cohorts, the DOPA-ApoEVs^CM^ and DOPA-ApoEVs/E7^CM^ groups exhibited elevated expression levels of OCN and RUNX2, with the DOPA-ApoEVs/E7^CM^ group showing the most pronounced increase (Fig. 6Q–T). Consistent with this, the expression levels of the osteogenic marker proteins RUNX2 and OCN in the E7 group were also substantially lower than those in the DOPA-ApoEVs/E7 group (Figure S21 F–I). Collectively, these findings demonstrate that surfaces modified with ApoEVs harvested from osteogenically differentiated BMSCs can effectively enhance indirect osteogenic differentiation within a DM inflammatory microenvironment through immunomodulatory mechanisms. To evaluate whether the modified surfaces could achieve effective osseointegration under diabetic inflammatory conditions, we further investigated their direct osteogenic potential. BMSCs were seeded directly onto the functionalized Ti discs to evaluate the impact of DOPA and ApoEV modification on osteoblastic activity within a simulated DM environment. After 7 days of culture, ALP staining was performed, followed by ARS staining on day 21. The results indicated that both the DOPA-ApoEVs^CM^ and DOPA-ApoEVs/E7^CM^ groups displayed enhanced ALP activity and increased calcium nodule deposition, with the DOPA-ApoEVs/E7^CM^ group showing a particularly prominent elevation (Fig. 6K). Our experimental findings demonstrate that the DOPA- and ApoEVs-co-modified directly facilitate osteogenic differentiation within a diabetic, inflammatory microenvironment. Notably, the DOPA^CM^ group exhibited enhanced osteogenic potential relative to the Ti^CM^ group, a phenomenon potentially attributable to the antioxidative functionalization afforded by DOPA. Furthermore, the capacity of the ApoEVs-E7 peptide to recruit BMSCs significantly strengthened the osteogenic performance of the modified surfaces under diabetic stress. These findings suggest that under diabetic conditions, although the E7 peptide can still promote osteogenesis by enhanced BMSC recruitment compared to the blank Ti control, this effect is heavily suppressed by the diabetic inflammatory microenvironment. While ApoEVs can ameliorate this diabetic inflammation and reverse macrophage polarization, their independent capacity for stem cell recruitment and osteogenesis remains limited. Taken together, our results demonstrate that ApoEVs and E7 exert a true synergistic effect, thereby significantly rescuing and enhancing osteogenic differentiation within the hostile diabetic inflammatory microenvironment. This suggests that immunomodulatory ApoEVs work in synergy with the E7 peptide to drive both direct and indirect osteogenic differentiation, ultimately strengthening osseointegration at the bone-implant interface.

### 2.7 Assessment of surface-mediated osteoimmunomodulation *in vivo*

**Fig. 7:**
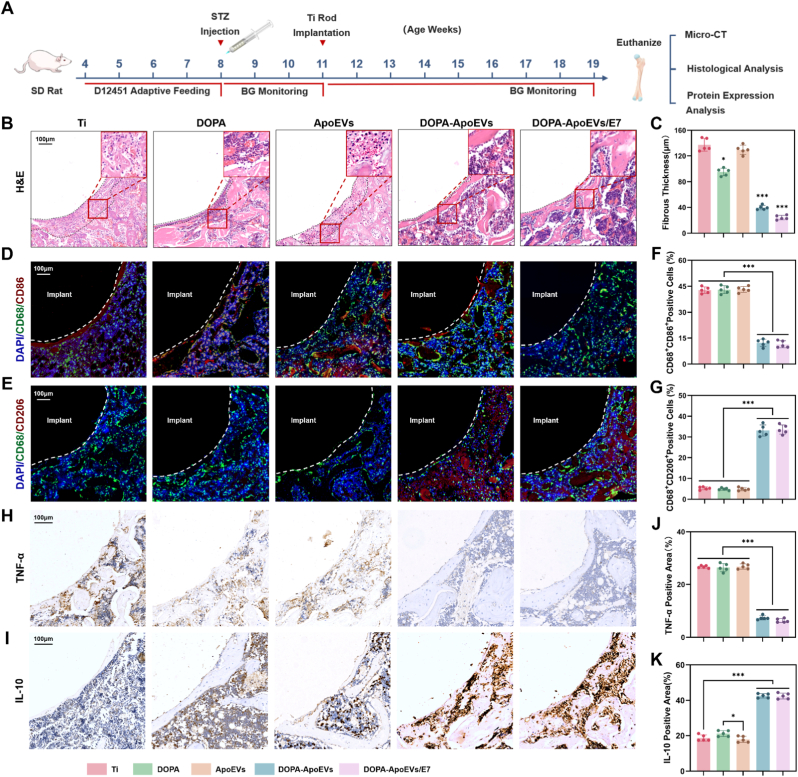
***In vivo* immunomodulatory effects of different modified surfaces in a DM model.** (A) Schematic representation of the animal modeling and experimental treatment workflow. (B, C) hematoxylin and eosin staining of the peri-implant tissues in the femurs of DM rats 1 week after implantation, accompanied by quantitative analysis of the fibrous capsule thickness (scale bar = 100 μm, n = 5). (D–G) Immunofluorescence staining evaluating the polarization state of macrophages surrounding the implants (green: macrophage marker cluster of differentiation (CD) 68; red: M1 marker CD86 and M2 marker CD206; blue: nuclei), along with corresponding quantitative analysis of the fluorescence signals (scale bar = 100 μm, n = 5). (H–K) Immunohistochemical staining assessing the expression of the pro-inflammatory marker tumor necrosis factor-α and the anti-inflammatory marker interleukin-10 in the peri-implant area, with quantitative results of the positive staining areas (scale bar = 100 μm, n = 5). Data are expressed as the mean ± standard deviation, with statistical analysis performed using one-way ANOVA and Tukey's post-hoc test. ∗p < 0.05, ∗∗p < 0.01, and ∗∗∗p < 0.001 indicate statistical significance.

Bone repair and regeneration are inextricably linked to angiogenesis. However, DM compromises this process through multiple pathological pathways. The persistent deposition of advanced glycation end products (AGEs) disrupts the vascular basement membrane, leading to a marked reduction in microvascular density. Such inadequate vascularization directly impairs osteogenesis, while the hyperglycemic environment independently suppresses osteoblast differentiation [55]. Furthermore, the nonenzymatic glycation of collagen increases the brittleness of the bone matrix, adversely affecting its biomechanical integrity [51]. Within this high-glucose milieu, the lineage commitment of BMSCs is altered; their capacity for osteogenic differentiation is diminished in favor of adipogenesis, resulting in reduced bone formation [56]. Chronic systemic hyperglycemia not only inhibits osteoblastic function but also accelerates bone resorption, ultimately culminating in decreased bone mineral density and compromised osseointegration [28]. To evaluate the immunomodulatory efficacy of the various modified titanium implants following femoral insertion in diabetic rats, we first established a streptozotocin (STZ)-induced DM model (Fig. 7A). Following induction, the rats exhibited classic polydipsia and polyuria, accompanied by a distinct ketotic odor in the urine. Successful establishment of the DM model was further verified using an impaired glucose tolerance test and persistent blood glucose levels ≥16.7 mM (Fig. S22A and B). In addition, we monitored the body weight of the rats; the results revealed that the diabetic (DM) rats exhibited an attenuated weight gain trajectory compared to the normal Control group, followed by a progressive weight loss starting from the second week(Fig. S22C). Furthermore, ELISA analysis demonstrated that the systemic levels of pro-inflammatory cytokines and the osteoclastogenic marker TRAP were significantly elevated in the DM group, whereas the key osteogenic marker OCN was markedly down-regulated(Fig. S22D and E). These cohesive findings further substantiate the robust and successful establishment of the DM rat model. Prior studies have documented that diabetes precipitates a cascade of skeletal complications, including osteoporosis and delayed defect healing; the chronic systemic inflammation triggered by prolonged hyperglycemia is increasingly recognized as a pivotal factor compromising prosthetic stability [49]. Given that macrophages exert their primary immunomodulatory influence during the nascent stages of healing, we implanted modified Ti rods into the femurs of diabetic rats to examine the local immune microenvironment (Fig. S23A–D). One week after implantation, the specimens were harvested for histological assessment. H&E staining demonstrated that the Ti, DOPA, and ApoEVs cohorts exhibited substantial fibrous capsule formation surrounding the prostheses, accompanied by a dense infiltration of inflammatory cells. Conversely, this peri-implant fibrous encapsulation was substantially attenuated in the DOPA-ApoEVs and DOPA-ApoEVs/E7 groups (Fig. 7B and C). Subsequently, we employed immunofluorescence staining to characterize the macrophage response within the peri-implant tissues. CD68 (green fluorescence) was used as a pan-macrophage marker, while CD86 and CD206 were co-labeled to differentiate between M1 and M2 phenotypes, respectively. The results indicated that the density of CD68^+^/CD86^+^ M1 macrophages surrounding the titanium rods in the DOPA-ApoEVs and DOPA-ApoEVs/E7 groups was markedly lower than that in the other cohorts. Conversely, these groups exhibited a significant enrichment of CD68^+^/CD206^+^ M2 macrophages (Fig. 7D–G). Immunohistochemical (IHC) staining further corroborated these findings; the DOPA-ApoEVs and DOPA-ApoEVs/E7 groups showed a significantly expanded area of IL-10-positive staining, whereas the Ti, DOPA, and ApoEVs groups were characterized by markedly larger TNF-α-positive regions (Fig. 7H–K). These results collectively demonstrate that within the physiological environment of diabetic rats, the DOPA-ApoEVs-modified surfaces exert potent anti-inflammatory effects by effectively facilitating the polarization of macrophages from an M1 to an M2 phenotype. While the STZ-induced rat model effectively mimics the hyperglycemic microenvironment characteristic of Type 1 diabetes, it is acknowledged that Type 2 Diabetes Mellitus (T2DM)—involving insulin resistance, chronic systemic inflammation, and metabolic syndrome—is far more prevalent among implant patients clinically. In a T2DM scenario, the compromised osseointegration is significantly exacerbated by a persistent pro-inflammatory microenvironment driven by chronic M1 macrophage polarization, alongside the accumulation of advanced glycation end-products (AGEs) that suppress bone formation. Notably, apoptotic cell-derived extracellular vesicles (ApoEVs) reprogramming M1 macrophages into the pro-healing M2 phenotype to resolve inflammation. Given that our functionalized ApoEVs coating maintains high stability and retention on Ti surfaces, we hypothesize that it could effectively mitigate the T2DM-associated chronic inflammation and restore the impaired osteo-immunological niche to promote bone-to-implant contact. Future studies utilizing specialized T2DM models will be pursued to comprehensively validate the clinical translational potential of this biomimetic coating. In conclusion, our evidence confirms that the developed DOPA- and ApoEVs-co-modified surfaces create a more favorable immune microenvironment under diabetic inflammatory conditions, thereby providing a robust foundation for enhanced bone regeneration and osseointegration.

### 2.8 Enhanced angiogenesis and osteogenesis promote *in vivo* osseointegration

**Fig. 8:**
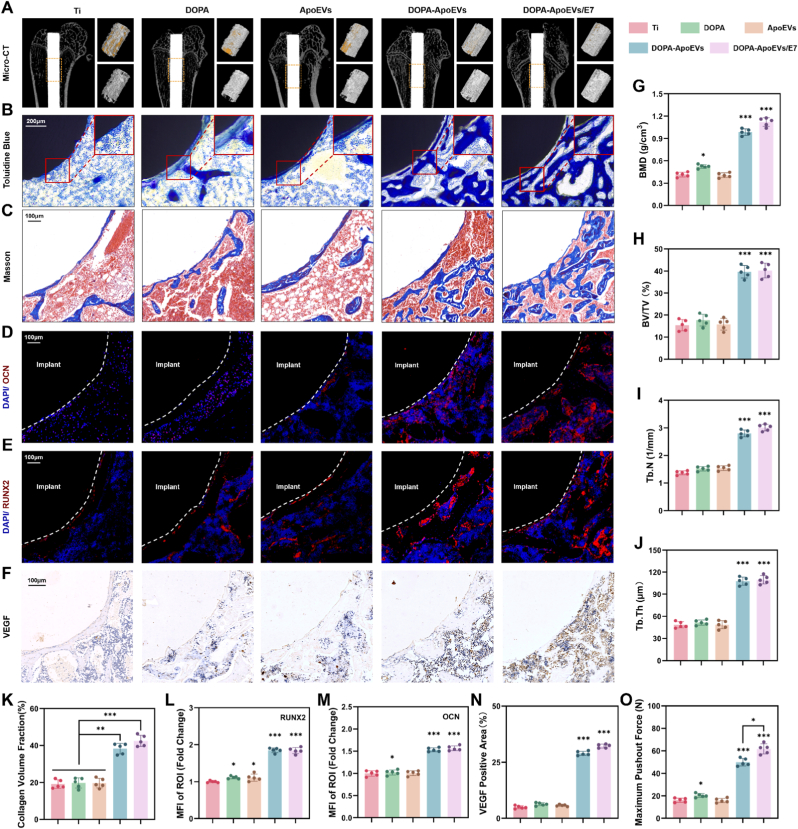
**3,4-dihydroxyphenylalanine-apoptotic extracellular vesicles/EPLQLKME7 (DOPA-ApoEVs/E7) -modified surfaces enhance *in vivo* osseointegration under DM conditions.** (A, G–J) Micro-CT scans and 3D-reconstructed images of femurs with various modified implants, featuring quantitative analysis of bone mineral density (BMD), bone volume/tissue volume (BV/TV), trabecular number (Tb.N, mm^−1^) and trabecular thickness (Tb.Th, μm) (n = 5). (B) Representative images of toluidine blue staining of the peri-implant bone tissue (scale bar = 200 μm). (C, K) Masson's trichrome staining of the peri-implant bone tissue (scale bar = 100 μm) and quantitative assessment of collagen formation (n = 5). (D–E, L–M) Immunofluorescence staining and quantitative analysis of the osteogenic hallmark proteins runt-related transcription factor 2 (RUNX2) and Osteocalcin (OCN) within the peri-implant area; red indicates RUNX2 or OCN, and blue indicates nuclei (scale bar = 100 μm, n = 5). F, N) Immunohistochemical staining evaluating the expression of the angiogenic marker vascular endothelial growth factor surrounding the implants, with corresponding quantitative analysis of the positive staining areas (scale bar = 100 μm, n = 5). Data are expressed as the mean ± standard deviation, with statistical analysis performed using one-way ANOVA and Tukey's post-hoc test. ∗p < 0.05, ∗∗p < 0.01, and ∗∗∗p < 0.001 indicate statistical significance.

Diabetes exerts a profound influence on bone turnover, skeletal morphology, and bone mineral density (BMD). Chronic systemic hyperglycemia suppresses osteoblastic function while simultaneously accelerating osteoclastic bone resorption, culminating in decreased BMD. In some clinical scenarios, even when bone density appears normal, the overall bone quality is compromised, significantly elevating the risk of osteoporosis [57]. Based on our *in vitro* findings, we confirmed that the functionalized surfaces achieved through the effective conjugation of DOPA and ApoEVs/E7 markedly enhanced angiogenesis and osteogenic capacity within a simulated diabetic inflammatory milieu. However, whether these modified surfaces can directly orchestrate the synergistic interplay between angiogenesis and osteogenesis to achieve stable osseointegration *in vivo* under diabetic conditions remains to be further validated. The STZ-induced DM model was established as previously described, after which the modified Ti rods were implanted into the rat femurs to observe osseointegration under diabetic conditions. We first evaluated the status of the bone-implant interface 8 weeks after implantation. Micro-CT scanning and three-dimensional (3D) reconstruction were performed on the femurs, revealing that the volume of newly formed bone surrounding the implants in the DOPA-ApoEVs and DOPA-ApoEVs/E7 groups was significantly higher than that in the other cohorts (Fig. 8A). Quantitative morphometric analysis further substantiated these observations; the bone mineral density (BMD) and bone volume fraction (BV/TV) in the peri-implant regions were substantially increased in the DOPA-ApoEVs and DOPA-ApoEVs/E7 groups. Additionally, these groups exhibited superior trabecular microarchitecture, characterized by increased trabecular number (Tb.N) and trabecular thickness (Tb.Th) (Fig. 8G–J). Subsequently, undecalcified bone sections were prepared and subjected to toluidine blue staining to evaluate the deposition of new bone surrounding the implants. The histological images demonstrated a significant increase in peri-implant bone formation within the DOPA-ApoEVs and DOPA-ApoEVs/E7 groups, characterized by a markedly higher bone-to-implant contact (BIC) ratio (Fig. 8B). Furthermore, Masson's trichrome staining was employed to assess the extent of collagen deposition around the titanium rods. Consistent with the collective evidence, the DOPA-ApoEVs and DOPA-ApoEVs/E7 cohorts exhibited substantially greater collagen density compared with the other groups, with the most pronounced accumulation observed in the DOPA-ApoEVs/E7 group (Fig. 8C–K). Biomechanical push-out testing serves as a critical method for quantifying the functional integrity of osseointegration (Figure S24 A). The maximum push-out force and interfacial shear strength analysis revealed that the DOPA-ApoEVs/E7 group possessed a significantly higher interfacial bonding strength compared to all other cohorts (Fig. 8O, Fig. S24B). Subsequently, immunofluorescence staining was performed to quantify the abundance of osteogenic markers within the peri-implant tissues. Quantitative analysis demonstrated that the fluorescence intensity and positive signal area for OCN, RUNX2, and Col1a1 proteins essential for osteoblastic differentiation were significantly elevated in the DOPA-ApoEVs and DOPA-ApoEVs/E7 groups. These findings substantiate that surfaces modified with DOPA-ApoEVs/E7 can more effectively drive *in vivo* osseointegration within a diabetic inflammatory microenvironment (Fig. 8D–E, L–M, and Fig. S25A–B). To conclude our histological analysis, IHC staining was conducted to evaluate the expression of proangiogenic markers *in vivo*. The analysis revealed a significant upregulation of VEGF in both the DOPA-ApoEVs and DOPA-ApoEVs/E7 cohorts (Fig. 8F–N). These findings provide robust evidence that surfaces functionalized with DOPA and ApoEVs/E7 possess a superior capacity to stimulate angiogenesis, even when subjected to the inhibitory pressures of a diabetic inflammatory microenvironment. Based on the aforementioned findings, we observed that the DOPA-ApoEVs and DOPA-ApoEVs/E7 groups exhibited a more effective integration between bone tissue and the implants, with the most pronounced results occurring in the DOPA-ApoEVs/E7 group. This outcome likely results from the synergistic interplay between osteogenesis and angiogenesis, an effect further amplified by the recruitment capacity of the E7 peptide. Notably, while ApoEVs have been previously recognized for their pro-osteogenic potential, our results confirm that ApoEVs without stable DOPA-mediated conjugation fail to effectively promote osseointegration under diabetic inflammatory conditions. This finding validates the fundamental role of the DOPA anchor in maintaining the bioactivity and localized delivery of the vesicles. It should be acknowledged as a limitation that an STZ-induced type 1 diabetes mellitus (T1DM) model was used in this study, whereas most clinical cases present with type 2 diabetes mellitus (T2DM). Nevertheless, both phenotypes share core pathological features at the implant interface, including persistent hyperglycemia and chronic inflammation that drives sustained M1 macrophage polarization. Crucially, while T2DM is characterized by systemic insulin resistance that exacerbates downstream inflammatory cascades, our localized DOPA-ApoEVs/E7 coating offers a distinct therapeutic advantage. Because ApoEVs regulate the local immune microenvironment via non-insulin-dependent pathways, their capacity to actively reprogram M1 macrophages into the pro-healing M2 phenotype remains highly effective even under conditions of systemic insulin resistance. By locally suppressing inflammation and subsequently activating the BMSC-recruiting capability of the E7 peptide, this bio-coating successfully bypasses systemic metabolic blockades to rescue impaired osseointegration. Collectively, these findings provide robust evidence that surfaces functionalized with DOPA and ApoEVs/E7 possess a superior capacity to stimulate angiogenesis, even when subjected to the inhibitory pressures of a diabetic inflammatory microenvironment. Based on the aforementioned findings, we observed that the DOPA-ApoEVs and DOPA-ApoEVs/E7 groups exhibited a more effective integration between bone tissue and the implants, with the most pronounced results occurring in the DOPA-ApoEVs/E7 group. This outcome likely results from the synergistic interplay between osteogenesis and angiogenesis, an effect further amplified by the recruitment capacity of the E7 peptide. Notably, while ApoEVs have been previously recognized for their pro-osteogenic potential, our results confirm that ApoEVs without stable DOPA-mediated conjugation fail to effectively promote osseointegration under diabetic inflammatory conditions. This finding validates the fundamental role of the DOPA anchor in maintaining the bioactivity and localized delivery of the vesicles.

The engineered “DOPA + ApoEVs + E7” coating overcomes the limitations conventional commercial implants. DOPA serves as a robust anchor that prevents rapid ApoEVs leakage, ensuring sustained stability to reprogram the macrophage-mediated diabetic inflammatory niche. Concurrently, the integrated E7 peptide rescues and accelerates the recruitment of endogenous mesenchymal stem cells, which is otherwise severely suppressed by diabetes. Compared to passive, osteoconductive commercial coatings that fail to withstand diabetic inflammation, this tripartite strategy orchestrates a dynamic, sequential cascade of early immune resolution, active stem cell recruitment, and enhanced immuno-angio-osteogenesis, offering a translationally superior solution for robust osseointegration under challenging diabetic pathologies. While the clinical promise of the “DOPA + ApoEVs + E7” coating is substantial, several translational limitations must be acknowledged. Currently in the laboratory stage, the fabrication process could be scaled up for industrial production through high-throughput centrifugation technologies. For preservation, the routine non-destructive storage of ApoEVs relies on −80 °C freezing or lyophilization; our laboratory data confirm that preserved ApoEVs effectively maintain their structural stability and biological functions after one year. Furthermore, while the therapeutic efficacy of this coating was validated using *in vitro* human cell models and *in vivo* rodent models, the clinical human diabetic microenvironment possesses far greater complexity, including systemic physical variations from long-term hyperglycemia and distinct degrees of peripheral vascular disease. Therefore, further evaluation in large animal models and subsequent clinical trials remains essential to fully substantiate the long-term safety and effectiveness of this synergistic coating before its widespread medical application. Collectively, we have demonstrated that the DOPA-ApoEVs/E7 system effectively enhances the coupling of angiogenesis and osteogenesis within the DM inflammatory microenvironment, thereby significantly strengthening the osseointegration of the implants.

## 3 Conclusion

Addressing the clinical bottleneck of poor osseointegration in Ti implants under DM inflammatory conditions, we designed a biomimetic surface modification strategy centered on ApoEVs, leading to the development of the DOPA-ApoEVs/E7-functionalized interface. The inherent immunomodulatory capacity of ApoEVs makes them ideal candidates for engineering bioactive bone implants that bridge osteogenesis with immune regulation. To achieve this, our study coupled mussel-inspired molecular adhesion with bio-orthogonal click chemistry. By utilizing a functional synthetic peptide containing both DOPA and azido groups as an intermediary, we established a stable biomimetic tethering system. This approach allowed for the mild yet highly efficient anchoring of ApoEVs onto the Ti alloy surface, resulting in the successful fabrication of the DOPA-ApoEVs/E7-functionalized surface while preserving the biological integrity of the vesicles. This surface capitalizes on the exceptional bioactivity of ApoEVs to markedly enhance peri-implant osseointegration. Our findings suggest that by precisely modulating the interfacial immune microenvironment and synergistically enhancing both osteogenesis and angiogenesis, the osseointegration capacity at the implant interface is significantly enhanced, demonstrating promising potential for clinical translation. Within inflammatory settings, the modified surface exhibits superior immunomodulatory capabilities, driving the polarization of M1 macrophages toward a pro-healing M2 phenotype. Transcriptomic sequencing analysis further confirms that DOPA-ApoEVs/E7-modified surfaces significantly downregulate the expression of pro-inflammatory cytokines and suppress the activation of multiple inflammation-pivotal signaling pathways. This optimized osteoimmune microenvironment establishes an ideal biological foundation for the osteogenic process, markedly enhancing osseointegration within the challenging DM inflammatory milieu. In summary, the surface modification strategy developed in this study is characterized by its simplicity, biocompatibility, and high efficiency. This modality provides a prospective therapeutic strategy to overcome the challenges of impaired implant osseointegration in the context of inflammatory diseases.

## 4 Experimental section

### 4.1 BMSC extraction and identification

BMSCs were isolated and harvested from the femoral and tibial marrow cavities of 6-week-old male SD rats under sterile conditions. Following anesthesia, the long bones of the lower limbs were cleared of adhering soft tissue. The bones were immersed in 75% ethanol for approximately 3 min and subsequently rinsed thoroughly with phosphate buffered saline. Both ends of the long bones were removed, and the marrow cavities were repeatedly flushed with culture medium using a 1 mL syringe until the effluent appeared clear. The resulting cell suspension was passed through a 70 μm cell strainer to eliminate debris. Red blood cell lysis buffer was added to the suspension, followed by centrifugation to remove erythrocytes. Upon reaching approximately 80% confluence, the cells were detached using 0.25% trypsin. BMSC purity was refined through successive medium changes during passage. To characterize the primary BMSCs, surface marker protein expression was evaluated using flow cytometry. Cells were identified as BMSCs based on the positive expression of CD29 (102207, Biolegend), CD44 (MA5-16908, ThermoScientific), and CD90 (202515, Biolegend) and the absence of the hematopoietic marker CD45 (202207, Biolegend). Data acquisition and subsequent analysis were performed using FlowJo 10 software (BD Biosciences, USA).

### 4.2 Apoptosis induction and ApoEVs isolation

At 12 h post-induction of apoptosis in osteogenically induced BMSCs, the extent of programmed cell death was quantified using a TUNEL Apoptosis Assay Kit (C1086, Beyotime, China). Simultaneously, we monitored the decrease in mitochondrial membrane potential using a mitochondrial membrane potential assay kit with JC-l (C2006, Beyotime, China). Following the 12 h apoptosis induction period, cells were harvested via trypsinization and processed using an Annexin V-FITC/PI Apoptosis Detection Kit (BD556547, BD Biosciences, USA). Apoptotic BMSCs were stained using FITC-labeled Annexin V and propidium iodide. Apoptotic populations were quantified using a BD FACSCanto™ II flow cytometer (BD Biosciences, USA), and the resulting data were processed and visualized using FlowJo 10 software (BD Biosciences, USA). To isolate apoptotic extracellular vesicles (ApoEVs), we collected the culture supernatant of apoptotic BMSCs and performed stepwise centrifugation: first at 800 × g at 4 °C for 10 min, then centrifuged at 2000 × g at 4 °C for 10 min to remove cell debris and collect the supernatant, and finally at 16,000 g at 4 °C for 30 min. This fraction was subjected to an additional 16,000 × g centrifugation cycle for further purification. Finally, the obtained ApoEVs were washed with filtered phosphate-buffered saline for subsequent use.

### 4.3 Characterization of ApoEVs

The concentration of isolated ApoEVs was quantified using a BCA protein assay Kit (NCM Biotech, China). To characterize the apoptotic cargo, WB analysis was performed to compare the protein profiles of ApoEVs with those of their parent cell lysates. The expression of key apoptosis-related markers was evaluated, including C1qa (A24519, ABclonal, China), Fas (A0233, ABclonal, China), and Caspase-3 (25128-1-AP, Proteintech, China). After the samples were diluted to a final concentration of approximately 4 × 10^7^ particles/mL, NTA was used to determine the size distribution and concentration of the ApoEVs. Additionally, the zeta potential was measured to assess the surface charge and stability of the vesicles. Morphological characterization was performed using TEM. A 20 μL aliquot of the ApoEVs solution was placed onto a TEM copper grid, and the droplet was allowed to remain on the grid for at least 1 min. The samples were then negatively stained with phosphotungstic acid solution for 1–10 min. After removing excess liquid with filter paper and air-drying the samples at room temperature, the morphology of the ApoEVs was observed using a transmission electron microscope at an accelerating voltage of 80.0 kV.

### 4.4 Ti surface functionalization

The experimental design was organized into five distinct groups: control (PBS only), DOPA group (DOPA-modified only), ApoEVs group (simple immersion in ApoEVs), DOPA-ApoEVs group (vesicle anchoring via click chemistry), and DOPA-ApoEVs/E7 group, in which the ApoEVs were further loaded with the E7 peptide. Peptides were synthesized using the Fmoc-based solid-phase peptide synthesis method. Specifically, a mussel-inspired ligand (DOPA)_6_-PEG_5_-DBCO, was designed to incorporate multiple DOPA units and a DBCO moiety. Additionally, a functionalized lipid-peptide conjugate, DSPE-PEG_2k_-E7, was synthesized with an azide modification. To minimize the oxidation of the catechol groups, each peptide solution was purged with nitrogen gas (N_2_) for 15 min before use. To conjugate the ApoEVs onto the Ti surface, the vesicles were first prepared by mixing DSPE-PEG_2k_-N_3_ and DSPE-PEG_2k_-E7 at a 1:1 wt ratio (RuixiBio, China) with the ApoEV suspension. Specifically, 2 × 10^12^ particles/mL ApoEVs were combined with 0.01 mg/mL DSPE-PEG_2k_-N_3_/E7 solution and allowed sufficient time for conjugation. For the substrates, 1 mm thick Ti discs (33 mm diameter) and 1 cm long Ti rods (1.5 mm diameter) were obtained from Baoji Titanium Industry Co. The discs were mirror-polished and the rod edges were smoothed before cleaning with acetone, 75% ethanol, and deionized water three times each, followed by high-temperature, high-pressure sterilization. The actual coating process involved two steps. First, the clean Ti substrates were submerged in a 0.01 mg/mL (DOPA)_6_-PEG_5_-DBCO solution (QYAOBIO, China) for 24 h to obtain a uniform base layer. For the click-chemistry groups, these DOPA-coated substrates were then transferred into a PBS solution containing 0.1 mg/mL modified ApoEVs for another 12 h of incubation. The simpler groups followed abbreviated versions of this protocol: the DOPA group terminated after the first 24 h, and the ApoEVs group was simply soaked in a vesicle solution. Finally, all prepared samples were rinsed thoroughly with Milli-Q ultrapure water, dried with N_2_, and stored at room temperature for subsequent experiments.

### 4.5 Material characterization

We first verified the quality of the synthesized building blocks. The mussel-inspired peptide (DOPA)_6_-PEG_5_-DBCO, was synthesized via the Fmoc solid-phase method and subsequently assessed for purity using HPLC (Agilent, USA). For the DSPE-conjugated components, specifically DSPE-PEG_2k_-N_3_ and DSPE-PEG_2k_-EPLQLKM, their molecular weights were confirmed through ESI-MS, and their chemical structures were characterized using ^l^H NMR. For the titanium surfaces, we evaluated how the modifications altered the surface topography. Surface morphology was characterized using a combination of SEM (FEI, USA) and AFM (Dimension ICON; Bruker, USA), which provided detailed visualization of the topographical shifts. To determine the chemical composition of these layers, EDS and XPS were used for elemental and functional group analyses. Because these modifications can alter surface interactions with liquids, hydrophobicity (or lack thereof) was measured using a Theta Lite contact angle meter (Biolin). Finally, the stability of ApoEV immobilization on the surface was assessed. To track this, the ApoEVs were labeled with PKH26 (C3637, Beyotime, China) before being conjugated onto the DOPA-primed Ti plates. After rigorous rinsing with Milli-Q water as a stability assessment, the remaining fluorescence was examined using a confocal microscope (Leica Stellaris 5, Germany). This allowed visualization of how effectively click chemistry retained the vesicles on the surface.

### 4.6 Cell culture

For the biological assays, RAW 264.7 cells and HUVECs were used, both sourced from the Shanghai Cell Bank of the Chinese Academy of Sciences. These, along with primary BMSCs, were seeded onto the various Ti surface modifications and maintained for durations of 24 h and 72 h depending on the specific experiment. The routine maintenance of these cells required tailored cultured media. BMSCs and MSCs were cultured in α-MEM (Gibco, USA) supplemented with 10% fetal bovine serum (FBS; Gibco, USA) and l% penicillin/streptomycin (P/S; NCM Biotech, China). RAW 264.7 cells were cultured in standard dulbecco's modified eagle medium (DMEM) (Gibco, USA) with 10% FBS and l% P/S. The HUVEC cells were cultured in endothelial cell culture medium (ECM; ScienCell, USA) containing 5% fetal bovine serum, l% endothelial cell growth factor, and l% P/S. All cultures were kept in a humidified incubator at 37 °C under a 5% CO_2_ atmosphere to ensure optimal growth conditions. For long-term storage, RAW 264.7 cells and HUVECs were preserved at −80 °C using a serum-free cryopreservation solution (NCM Biotech, China).

### 4.7 Biocompatibility assessments

To evaluate how our modified Ti surfaces interacted with the cellular environment, RAW 264.7 cells, BMSCs, and HUVECs were seeded across the different groups. The three cell types were initially maintained under standard culture conditions. Subsequently, the medium was replaced with conditioned medium (DM-CM) designed to simulate a diabetic microenvironment, supplemented with 25 mM high glucose and 100 ng/mL LPS. After 24 h of co-culture, a live/dead assay (Beyotime Biotechnology, China) was performed. This provided visual confirmation of viability, with Calcein-AM staining living cells green and dead cells stained with propidium iodide (PI) red. To support these findings with quantitative data on membrane integrity, an LDH cytotoxicity assay (Beyotime Biotechnology, China) was also performed. Cell proliferation over 72 h. Using a standard CCK-8 protocol (NCM Biotech, China), the growth media was replaced with a 10% CCK-8 solution at the 24- and 72-h time points. Following 1 h of incubation at 37 °C, protected from light, the plates were read at 450 nm using a Thermo Fisher microplate reader. The cytoskeletal organization and morphology of BMSCs and HUVECs were visualized via phalloidin staining. After 24 h of culture on various modified surfaces, 4% paraformaldehyde (NCM Biotech, China) was used to fix the cells for 20 min. The actin filaments were stained with iFluor 488-conjugated phalloidin (Yeasen, China) at room temperature in the dark for 1 h, followed by nuclear counterstaining with 4′,6-diamidino-2-phenylindole (DAPI; Beyotime Biotechnology, China) for 10 min. Images were acquired using a confocal laser scanning microscope (Leica Stellaris 5, Germany) to evaluate cell adhesion and spreading. Semi-quantitative analysis of the morphological parameters was performed using ImageJ software (version 1.54d).

### 4.8 Macrophage polarization assay

To evaluate the immunomodulatory effects of ApoEVs/E7-modified Ti surfaces, RAW264.7 macrophages were seeded onto the substrates at a density of 2 × 10^5^ cells/well and cultured in DMEM supplemented with 25 mM high glucose and 100 ng/mL LPS to simulate the diabetic inflammatory microenvironment. After 48 h, the culture supernatants were harvested to quantify the secretion of TNF-α, IL-10, and VEGF using commercial enzyme-linked immunosorbent assay (ELISA) kits (Multi Sciences, EK282EG, EK210EGA, and EK183EGA). For phenotypic visualization, cells were fixed in 4% paraformaldehyde, permeabilized with 0.1% Triton X-100, and blocked before overnight incubation at 4 °C with primary antibodies against iNOS (1:200, ab178945) and Arg-1 (1:200, ab91279). Subsequent labeling was performed using Alexa Fluor 647-conjugated secondary antibodies and iFluor 488-phalloidin (1:1000, Yeasen) for 1 h at 37 °C in the dark, after which nuclei were stained with 4′,6-diamidino-2-phenylindole and imaged via confocal microscopy (Leica Stellaris 5). To further quantify polarization, 5 × 10^5^ cells were collected, centrifuged (4 °C, 300 × g, 5 min), and resuspended in PBS for staining with antibodies against CD11b, CD86, and CD206 (Biolegend, USA). These subpopulations were analyzed using a BD FACSCanto™ II flow cytometer (BD Biosciences, USA), with data processed using FlowJo v10. Finally, the expression levels of M1-and M2-related proteins and genes were assessed through WB and RNA extraction, respectively, to verify the shifts in macrophage lineage commitment.

### 4.9 *In vitro* osteogenic differentiation

To evaluate the *in vitro* osteogenic potential of the modified surfaces, macrophage- CM was prepared by harvesting supernatants from RAW264.7 cells cultured on various Ti substrates. This medium was mixed in a 1:1 ratio with low-glucose DMEM and supplemented with 25 mM high glucose and 100 ng/mL LPS to replicate the DM inflammatory microenvironment. To initiate osteogenic induction, the CM was further enriched with 10^−4^ mM dexamethasone (Sigma-Aldrich, USA), 5 × 10^−2^ mg/mL ascorbic acid (Sigma-Aldrich, USA), and 10 mM β-glycerophosphate (Sigma-Aldrich, USA). BMSCs were then isolated and subjected to both direct and indirect osteogenic challenges; specifically, cells were either seeded onto the modified Ti plates or cultured in standard culture dishes. After an initial 36 h equilibration in low-glucose DMEM containing 10% FBS, the DMEM media were replaced with the prepared osteogenic CM and refreshed every 2–3 days. Osteogenic markers were assessed at multiple time points: following 48 h of incubation, the expression levels of RUNX2 and OCN (l:200, Abcam, UK) were evaluated via immunofluorescence and confocal laser scanning microscopy (Leica Stellaris 5), while total RNA was extracted for RT-qPCR, and protein was harvested for downstream Western blot analysis. Early-stage osteogenesis was quantified at day 7 through ALP staining and activity assays (Beyotime, China), while terminal mineralization was stained and evaluated on day 21 using Alizarin Red S (ARS) (OriCell®, Cyagen Biosciences, USA), followed by semi-quantitative analysis using perchloric acid dissolution. Accordingly, we performed the ALP on day 7 and ARS staining on day 21 for both the cells grown directly on the Ti surfaces and those treated indirectly with the CM to ensure a comprehensive evaluation of the surfaces' osteoinductive capacity.

### 4.10 *In vitro* angiogenesis

To investigate the potential of various surface treatments to construct a proangiogenic immune microenvironment, thereby indirectly enhancing vascularization, macrophage-conditioned media (CM) were prepared by blending supernatants from RAW264.7 cells cultured on the respective Ti substrates with fresh ECM medium in a 1:1 ratio. The angiogenic potential was first assessed via a wound-healing assay; HUVECs were seeded and grown to confluence in ECM, at which point a uniform scratch was introduced across the diameter of each well. The cells were then transitioned to the specific CM groups, and the progression of scratch closure was monitored at 4 h and 24 h post-wounding via calcein-AM staining and inverted fluorescence microscopy (Leica, Germany). To further quantify vertical chemotactic capacity, a Transwell migration assay (Corning, USA) was employed. HUVECs were placed in the upper chambers, while the lower compartments were filled with the corresponding CM; following a 24 h incubation, migrated cells were fixed and stained with 0.1% crystal violet (Beyotime, China) for microscopic visualization. The capacity for capillary-like network formation was evaluated using a tube formation assay. Matrigel was mixed with DMEM at a 2:1 ratio, and 40 μL of this mixture was polymerized in each well of a 24-well plate at 37 °C for 1 h. HUVECs were then seeded onto the gel at a density of 2 × 10^5^/well in the presence of CM. Tubular structures were visualized at 4 h and 24 h using calcein-AM staining. Finally, to corroborate these functional observations at the molecular level, HUVECs were harvested after 48 h of CM stimulation for total RNA and protein extraction, followed by Western blotting and RT-qPCR to analyze the expression of proangiogenic markers.

### 4.11 RT-qPCR

Total RNA was harvested from the variously treated cells using TRIzol reagent (15596018CN, Invitrogen), after which complementary DNA (cDNA) was synthesized via reverse transcription using the PrimerScript™ RT Master Mix Kit (RR036A, Takara Bio Inc). The resulting cDNA templates were amplified with TB Green Premix Ex *Taq*II (RR820A, Takara Bio Inc.) on a Roche LightCycler 480 II real-time PCR system. Oligonucleotide sequences for target genes are provided in Supplementary Table 1.

### 4.12 Western blotting

For protein analysis, total cellular protein was extracted using RIPA lysis buffer (Beyotime, China), and concentrations were standardized with a BCA protein assay kit (NCM Biotech, China). Equal amounts of protein were separated via sodium dodecyl sulfate–polyacrylamide gel electrophoresis at 120 V for 90 min and subsequently transferred onto polyvinylidene fluoride membranes at 250 mA for another 90 min. Following a 1 h blocking step, the membranes were incubated overnight at 4 °C with primary antibodies against iNOS (ab178945, Abcam), Arg-1 (ab91279, Abcam), VEGF (A12303, ABclonal), CD31 (A19014, ABclonal), Col1a1 (A22090, ABclonal), OCN (A20800, ABclonal), RUNX2 (20700-l-AP, Proteintech), SP7 (28694-l-AP, Proteintech), STAT3(60199-1-Ig, Proteintech), p-STAT3(60479-1-Ig), p65(80979-1-RR, Proteintech), p-p65(82335-1-RR, Proteintech) and CXCL1 (12335-1-AP, Proteintech). After thorough washing with TBST, the membranes were incubated with appropriate secondary antibodies at room temperature for 1 h prior to chemiluminescent detection.

### 4.13 ROS

Intracellular reactive oxygen species (ROS) levels in macrophages were evaluated using a commercial ROS assay kit (S0034S, Beyotime, China) according to the manufacturer's instructions. Briefly, the DCFH-DA probe was diluted with the probe dilution buffer at a ratio of 1:1000 to achieve a final working concentration of 10 μM. After removing the culture medium, an appropriate volume of the diluted DCFH-DA solution was added to the cells, followed by incubation in a humidified incubator at 37 °C for 20 min. Subsequently, the cells were washed three times with phosphate-buffered saline (PBS) to thoroughly eliminate extracellular, non-internalized probes. The intracellular fluorescence intensity was immediately visualized and captured using a laser scanning confocal microscope (Leica Stellaris 5).

### 4.14 Animal models

All animal procedures were performed in strict accordance with the guidelines for the care and use of laboratory animals and were formally approved by the Ethics Committee of Soochow University (approval No. SUDA20251229A07). Male SD rats were obtained from the Laboratory Animal Center of Soochow University. To establish the DM model, 5-week-old rats (initial weight, 100 g) were maintained on a high-fat diet (D12451) for 4 weeks until reaching a weight range of 240–280 g. Following a 12 h fast, a freshly prepared 1% (w/v) streptozotocin (STZ; Sigma-Aldrich) solution in citrate buffer (pH 4.2–4.5) was administered via intraperitoneal injection at a dosage of 35 mg/kg. Successful induction of DM was confirmed by non-fasting blood glucose levels ≥16.7 mM, monitored daily via tail vein blood sampling. Pancreatic islet function was further characterized through a glucose tolerance test (GTT); after a 12-h fast, rats received an intraperitoneal glucose bolus (1 g/kg), with blood glucose fluctuations recorded at 30, 60, 90, and 120 min post-injection. The body weight of the DM rats were monitored and recorded daily throughout the experimental period to assess the progression of the diabetic model. Rat serum levels of OCN, TRAP, TNF-α, and IL-6 were determined using commercial ELISA kits according to the manufacturers' instructions: OCN (CSB-E05129N, CUSABIO, China), TRAP (T775190-96 T/EA, MACKLIN, China), TNF-α (EK382EGB, Multi Sciences, China), and IL-6 (EK306HS, Multi Sciences, China). Animals were randomly assigned to five experimental cohorts based on the Ti implant surface modifications: control, DOPA, ApoEVs, DOPA-ApoEVs, and DOPA-ApoEVs/E7. Following systemic anesthesia induced via an intraperitoneal injection of pentobarbital (3.5 mg/kg), the rats were prepared for the surgery. Under sterile conditions, a midline incision was made at the knee joint to expose the distal femur. Titanium rods (10 mm in length, 1.5 mm in diameter) with the respective surface treatments were implanted vertically into the femoral intercondylar notch. Postoperative blood glucose levels were monitored weekly to ensure the persistence of the diabetic state. At 8 weeks after implantation, all rats were euthanized, and the bilateral femora, along with major organs including the heart, liver, spleen, lungs, and kidneys, were harvested for subsequent histological and systemic evaluation.

### 4.15 Micro-CT analysis and biomechanical pull-out test

Following euthanasia, the harvested femora were meticulously stripped of adherent soft tissues and immediately stabilized in 10% neutral buffered formalin for 12 h. To evaluate peri-implant bone regeneration, the specimens were scanned using a SkyScan 1176 Micro-CT system (Bruker, Belgium) with the following acquisition parameters: a voltage of 50 kV, a 500 μA electric current, and a spatial resolution of 18 μm per slice. The scanning field was set to 2 cm × 2 cm with a rotation step of 0.7°. Volumetric reconstruction was performed using NRecon, while the quantitative assessment of bone morphometry was conducted using CTAn. Specifically, the region of interest surrounding the implant was analyzed for bone mineral density (BMD, mg/cm^3^), bone volume/total volume (BV/TV, %), trabecular number (Tb.N, 1/mm), and trabecular thickness (Tb.Th, μm) to compare osseointegration across the experimental cohorts. Representative three-dimensional visualizations were generated using Avizo software (Thermo Scientific, USA). To determine the functional strength of the bone-implant interface, the femurs were subjected to a biomechanical pull-out test using a universal material testing system (HY1080, China). The specimens were securely embedded in bone cement onto the device base to ensure stability. A push-out test was then performed by applying a load along the longitudinal axis of the femur at a constant displacement rate of 1 mm/min. Interfacial bonding strength was quantified by recording the maximum push-out force and interfacial shear strength encountered during the testing process.

### 4.16 Exploration of immunoregulatory mechanisms

For histological assessment, five femoral specimens containing Ti implants were initially dehydrated and embedded in polymethylmethacrylate resin. The resulting resin blocks were processed into high-quality undecalcified sections and subsequently stained with toluidine blue to visualize new bone formation at the implant-bone interface. The remaining femoral samples were fixed in 10% neutral buffered formalin for 48 h and subsequently decalcified in 10% ethylenediaminetetraacetic acid (EDTA; Sigma-Aldrich) for 4–5 weeks. Once decalcification was complete, the Ti rods were carefully pulled out, and the residual bone tissues were embedded in paraffin and sectioned for downstream staining protocols. Following deparaffinization and rehydration, H&E staining was performed to evaluate peri-implant fibrous hyperplasia, while Masson's trichrome staining was employed to assess collagen deposition levels surrounding the original implant site. For IHC analysis, sections underwent heat-induced antigen retrieval and blocking prior to incubation with antibodies against TNF-α (GB11188, Servicebio, China), IL-10 (GB11534, Servicebio, China), and VEGF (GB15165, Servicebio, China) to identify differences in local inflammatory and angiogenic profiles. To further elucidate macrophage polarization and osteogenic activity in situ, immunofluorescence staining was conducted. Macrophages were identified using CD68 (Abcam, ab201340), with CD86 (13395-l-AP, Proteintech) and CD206 (18704-1-AP, Proteintech) serving as markers for M1 and M2 phenotypes, respectively. Osteogenic differentiation was characterized by staining for RUNX2 (20700-1-AP, Proteintech), OCN (A20800, ABclonal), and Col1a1 (A22090, ABclonal). All fluorescently labeled sections were visualized via confocal laser scanning microscopy, and the resulting images were processed for quantitative fluorescence intensity analysis using ImageJ software.

### 4.17 RNA-seq analysis

To gain deeper insights into the transcriptomic shifts induced by the modified surfaces, total RNA was isolated from RAW 264.7 cells after a 2-day culture period on either the control Ti or DOPA-ApoEVs/E7 surfaces. The extraction was performed using TRIzol reagent (15596018CN, Invitrogen), followed by a rigorous purification process. The concentration, purity, and structural integrity of the harvested RNA were verified using an Agilent 2100 Bioanalyzer prior to library construction. Subsequently, cDNA libraries were prepared utilizing the VAHTS Universal V6 RNA-seq Library Prep Kit. High-throughput sequencing and the ensuing bioinformatic analyses were conducted by OE Biotech Co., Ltd. (Shanghai, China) to identify differentially expressed genes and enriched biological pathways.

### 4.18 Statistical analysis

Quantitative data are expressed as the mean ± standard deviation (SD). Statistical analyses were conducted using GraphPad Prism (version 9.0.0), where the selection of the analytical method was dictated by the experimental design. Specifically, an independent Student's *t*-test was used for comparisons between two distinct groups, whereas, for analyses involving more than two cohorts, statistical significance was determined using one-way analysis of variance or two-way analysis of variance. In all graphical representations, the error bars denote the standard deviation. A value of p < 0.05 was prespecified as the threshold for considering a result statistically significant.

## Ethics approval and consent to participate

All animal procedures were performed in strict accordance with the guidelines for the Care and Use of Laboratory Animals and were formally approved by the Ethics Committee of Soochow University (Approval No.SUDA20251229A07). Male Sprague-Dawley (SD) rats were obtained from the Laboratory Animal Center of Soochow University. All the rats were housed and euthanized in compliance with the animal research policies of the National Ministry of Health as per standard regulations.

## CRediT authorship contribution statement

**Jining Shen:** Data curation, Investigation, Methodology, Software, Writing – original draft, Writing – review & editing. **Gaoran Ge:** Formal analysis, Methodology, Validation, Visualization, Writing – review & editing. **Tianpeng Xu:** Investigation, Methodology, Writing – review & editing. **Qiufei Wang:** Investigation, Methodology. **Yi Qin:** Formal analysis, Methodology. **Xiaoheng Lu:** Data curation, Methodology. **Jin Liu:** Project administration. **Pengcheng Xu:** Data curation, Formal analysis, Methodology. **Kefan Wu:** Methodology, Validation. **Fan Liu:** Funding acquisition, Project administration, Resources, Supervision. **Xing Yang:** Conceptualization, Data curation, Project administration. **Dechun Geng:** Conceptualization, Project administration, Writing – original draft, Writing – review & editing. **Yake Liu:** Conceptualization, Funding acquisition, Supervision, Writing – original draft, Writing – review & editing.

## Declaration of competing interest

The authors declare no conflict of interest.

## References

[1] Bruns S., Krüger D., Galli S., Wieland D.C.F., Hammel J.U., Beckmann F., Wennerberg A., Willumeit-Römer R., Zeller-Plumhoff B., Moosmann J. (2023). On the material dependency of peri-implant morphology and stability in healing bone. Bioact. Mater..

[2] Shen D., Li Y., Shi J., Zhang T., Nie J.-J., Chen D., Xia D., Zheng Y. (2025). Biodegradable Zn-Li-Mn alloy to achieve optimal strength and ductility for bone implants. Acta Biomater..

[3] Su Y., Fu J., Lee W., Du S., Qin Y.-X., Zheng Y., Wang Y., Zhu D. (2022). Improved mechanical, degradation, and biological performances of Zn–Fe alloys as bioresorbable implants. Bioact. Mater..

[4] Liu A., Lin D., Zhao H., Chen L., Cai B., Lin K., Shen S.G. (2021). Optimized BMSC-derived osteoinductive exosomes immobilized in hierarchical scaffold via lyophilization for bone repair through Bmpr2/Acvr2b competitive receptor-activated Smad pathway. Biomaterials.

[5] Xiang Y., Zhuge P., Qi X., Ge X., Xiang J., Xu H., Cai E., Lan Y., Chen X., Li Y., Shi Y., Shen J., Liu J. (2024). A cuttlefish ink nanoparticle-reinforced biopolymer hydrogel with robust adhesive and immunomodulatory features for treating oral ulcers in diabetes. Bioact. Mater..

[6] Qi X., Li Y., Xiang Y., Chen Y., Shi Y., Ge X., Zeng B., Shen J. (2025). Hyperthermia-enhanced immunoregulation hydrogel for oxygenation and ROS neutralization in diabetic foot ulcers. Cell Biomater..

[7] Hogrebe N.J., Ishahak M., Millman J.R. (2023). Developments in stem cell-derived islet replacement therapy for treating type 1 diabetes. Cell Stem Cell.

[8] De Oliveira P.G.F.P., Bonfante E.A., Bergamo E.T.P., De Souza S.L.S., Riella L., Torroni A., Benalcazar Jalkh E.B., Witek L., Lopez C.D., Zambuzzi W.F., Coelho P.G. (2020). Obesity/Metabolic syndrome and diabetes mellitus on peri-implantitis. Trends Endocrinol. Metabol..

[9] Bai L., Feng M., Zhang Q., Cai Z., Li Q., Li Y., Ma C., Xiao J., Lin Y. (2024). Synergistic osteogenic and antiapoptotic framework nucleic acid complexes prevent diabetic osteoporosis. Adv. Funct. Mater..

[10] Juin S.K., Pushpakumar S., Sen U. (2023). Alpha-glucosidase inhibitor mitigates bone loss in type-1 diabetes. Physiology.

[11] Wu J., Chen M., Xiao Y., Yang H., Wang G., Zhang X., Dai L., Yuan Z. (2024). The bioactive interface of titanium implant with both anti‐oxidative stress and immunomodulatory properties for enhancing osseointegration under diabetic condition. Adv. Healthcare Mater..

[12] He M., Wang H., Han Q., Shi X., He S., Sun J., Zhu Z., Gan X., Deng Y. (2023). Glucose-primed PEEK orthopedic implants for antibacterial therapy and safeguarding diabetic osseointegration. Biomaterials.

[13] Louiselle A.E., Niemiec S.M., Zgheib C., Liechty K.W. (2021). Macrophage polarization and diabetic wound healing. Transl. Res..

[14] Shi B., Phan T.K., Poon I.K.H. (2025). Extracellular vesicles from the dead: the final message. Trends Cell Biol..

[15] Hardy M.-P., Audemard É., Migneault F., Feghaly A., Brochu S., Gendron P., Boilard É., Major F., Dieudé M., Hébert M.-J., Perreault C. (2019). Apoptotic endothelial cells release small extracellular vesicles loaded with immunostimulatory viral-like RNAs. Sci. Rep..

[16] Gao Q., Wang L., Wang S., Huang B., Jing Y., Su J. (2022). Bone marrow mesenchymal stromal cells: identification, classification, and differentiation. Front. Cell Dev. Biol..

[17] Wang Y., Fang J., Liu B., Shao C., Shi Y. (2022). Reciprocal regulation of mesenchymal stem cells and immune responses. Cell Stem Cell.

[18] Tomlinson R.E., Silva M.J. (2013). Skeletal blood flow in bone repair and maintenance. Bone Res..

[19] Tian G., Yin H., Zheng J., Yu R., Ding Z., Yan Z., Tang Y., Wu J., Ning C., Yuan X., Liao C., Sui X., Zhao Z., Liu S., Guo W., Guo Q. (2024). Promotion of osteochondral repair through immune microenvironment regulation and activation of endogenous chondrogenesis via the release of apoptotic vesicles from donor MSCs. Bioact. Mater..

[20] Shi P., Gao H., Cheng Z., Wu W., Zhang A., Chen X., Wu W., Zhang Y. (2025). USP5‐Rich apoptotic extracellular vesicles regulate nucleus pulposus cells apoptosis and DNA damage repair by preventing E2F1 proteasomal degradation. J. Extracell. Vesicles.

[21] Li D., Zhu B., Jiao W., Chen W., Zhang X., Li D., Zhao W., Ding Y., Zheng G., Zhao S., Pan T., Rong Y., Yu H. (2026). Apoptotic extracellular vesicles derived from UC-MSCs promote spinal cord injury repair by inhibiting ferroptosis via OTUD1. Free Radic. Biol. Med..

[22] Cheng Y., Zhu Y., Liu Y., Liu X., Ding Y., Li D., Zhang X., Liu Y. (2024). Tailored apoptotic vesicles promote bone regeneration by releasing the osteoinductive brake. Int. J. Oral Sci..

[23] Zhao Q., Lu B., Qian S., Mao J., Zhang L., Zhang Y., Mao X., Cui W., Sun X. (2024). Biogenerated oxygen‐related environmental stressed apoptotic vesicle targets endothelial cells. Adv. Sci..

[24] Pan G., Li B. (2023). A dynamic biointerface controls mussel adhesion. Science.

[25] Sivasundarampillai J., Youssef L., Priemel T., Mikulin S., Eren E.D., Zaslansky P., Jehle F., Harrington M.J. (2023). A strong quick-release biointerface in mussels mediated by serotonergic cilia-based adhesion. Science.

[26] Bai J., Wang H., Chen H., Ge G., Wang M., Gao A., Tong L., Xu Y., Yang H., Pan G., Chu P.K., Geng D. (2020). Biomimetic osteogenic peptide with mussel adhesion and osteoimmunomodulatory functions to ameliorate interfacial osseointegration under chronic inflammation. Biomaterials.

[27] Wang T., Bai J., Lu M., Huang C., Geng D., Chen G., Wang L., Qi J., Cui W., Deng L. (2022). Engineering immunomodulatory and osteoinductive implant surfaces via mussel adhesion-mediated ion coordination and molecular clicking. Nat. Commun..

[28] Ge G., Wang W., Wang Q., Wang M., Wang T., Yu L., Zhang X., Zhu C., Xu Y., Yang H., Bai J., Pan G., Geng D. (2024). Extracellular vesicle clicking on osteoimplants through biomimetic molecular adhesion enables immune‐enhanced osseointegration in diabetics. Adv. Funct. Mater..

[29] Yang Z., Zhao X., Hao R., Tu Q., Tian X., Xiao Y., Xiong K., Wang M., Feng Y., Huang N., Pan G. (2020). Bioclickable and mussel adhesive peptide mimics for engineering vascular stent surfaces. Proc. Natl. Acad. Sci. USA.

[30] Bai J., Ge G., Wang Q., Li W., Zheng K., Xu Y., Yang H., Pan G., Geng D. (2022). Engineering stem cell recruitment and osteoinduction via bioadhesive molecular mimics to improve osteoporotic bone-implant integration. Research.

[31] Kang Y., Han X., Zhou S., Wang X., Wang Y., Song P., Su X., Qin M., Qian D., Meng H., Yan J., Pu F., Zhang H. (2026). Engineered apoptotic extracellular vesicles for programmable regulation of Neutrophil‐Macrophage‐ROS pathogenic axis to reconstruct rheumatoid arthritis microenvironment. Adv. Mater..

[32] Horwitz E.M., Le Blanc K., Dominici M., Mueller I., Slaper-Cortenbach I., Marini F.C., Deans R.J., Krause D.S., Keating A. (2005). Clarification of the nomenclature for MSC: the international society for cellular therapy position statement. Cytotherapy.

[33] Lei F., Huang Z., Ou Q., Li J., Liu M., Ma L., Tan L., Lin Z., Kou X. (2023). Apoptotic vesicles rejuvenate mesenchymal stem cells via Rab7-mediated autolysosome formation and alleviate bone loss in aging mice. Nano Res..

[34] Damiati L.A., Tsimbouri M.P., Hernandez V.-L., Jayawarna V., Ginty M., Childs P., Xiao Y., Burgess K., Wells J., Sprott M.R., Meek R.M.D., Li P., Oreffo R.O.C., Nobbs A., Ramage G., Su B., Salmeron-Sanchez M., Dalby M.J. (2022). Materials-driven fibronectin assembly on nanoscale topography enhances mesenchymal stem cell adhesion, protecting cells from bacterial virulence factors and preventing biofilm formation. Biomaterials.

[35] Li J., Zhao J., Xu Y., Xu A., He F. (2023). Titanium surface interacting with blood clot enhanced migration and osteogenic differentiation of bone marrow mesenchymal stem cells. Front. Bioeng. Biotechnol..

[36] Klein M.O., Bijelic A., Ziebart T., Koch F., Kämmerer P.W., Wieland M., Konerding M.A., Al‐Nawas B. (2013). Submicron scale‐structured hydrophilic titanium surfaces promote early osteogenic gene response for cell adhesion and cell differentiation. Clin. Implant Dent. Relat. Res..

[37] Huang C.-H., Yoshimura M. (2023). Biocompatible hydroxyapatite ceramic coating on titanium alloys by electrochemical methods via growing integration layers [GIL] strategy: a review. Ceram. Int..

[38] Sheikh K.A., Khan M.M., Dey A., Wani M.F. (2025). Advancements in surface engineering: a comprehensive review of functionally graded coatings for biomaterial implants. Results Surf. Interfaces.

[39] Pecherskaya E., Golubkov P., Konovalov S., Gurin S., Novichkov M. (2024). Mechanism, models, and influence of heterogeneous factors of the microarc oxidation process: a comprehensive review. Rev. Adv. Mater. Sci..

[40] Popova A.D., Sheveyko A.N., Kuptsov K.A., Advakhova D.Yu, Karyagina A.S., Gromov A.V., Krivozubov M.S., Orlova P.A., Volkov A.V., Slukin P.V., Ignatov S.G., Shubina I.Zh, Ilnitskaya A.S., Gloushankova N.A., Timoshenko R.V., Erofeev A.S., Shtansky D.V. (2023). Osteoconductive, osteogenic, and antipathogenic plasma electrolytic oxidation coatings on titanium implants with BMP-2. ACS Appl. Mater. Interfaces.

[41] Xu N., Fu J., Zhao L., Chu P.K., Huo K. (2020). Biofunctional elements incorporated nano/microstructured coatings on titanium implants with enhanced osteogenic and antibacterial performance. Adv. Healthcare Mater..

[42] Jurczak P., Witkowska J., Rodziewicz-Motowidło S., Lach S. (2020). Proteins, peptides and peptidomimetics as active agents in implant surface functionalization. Adv. Colloid Interface Sci..

[43] Bhatt H.B., Smith R.J. (2015). Fatty liver disease in diabetes mellitus. Hepatobiliary Surg. Nutr..

[44] Ferguson D., Finck B.N. (2021). Emerging therapeutic approaches for the treatment of NAFLD and type 2 diabetes mellitus. Nat. Rev. Endocrinol..

[45] Simoes I.C.M., Janikiewicz J., Bauer J., Karkucinska-Wieckowska A., Kalinowski P., Dobrzyń A., Wolski A., Pronicki M., Zieniewicz K., Dobrzyń P., Krawczyk M., Zischka H., Wieckowski M.R., Potes Y. (2019). Fat and sugar—A dangerous duet. A comparative review on metabolic remodeling in rodent models of nonalcoholic fatty liver disease. Nutrients.

[46] Xiang G., Liu K., Wang T., Hu X., Wang J., Gao Z., Lei W., Feng Y., Tao T.H. (2021). In situ regulation of macrophage polarization to enhance osseointegration under diabetic conditions using injectable silk/sitagliptin gel scaffolds. Adv. Sci..

[47] Zhou W., Liu Y., Nie X., Zhu C., Xiong L., Zhou J., Huang W. (2025). Peptide-based inflammation-responsive implant coating sequentially regulates bone regeneration to enhance interfacial osseointegration. Nat. Commun..

[48] Su C.-F., Chen Y.-F., Tsai Y.-J., Weng S.-M., Jan J.-S. (2021). Antioxidant activity of linear and star-shaped polypeptides modified with dopamine and glutathione. Eur. Polym. J..

[49] Liu L., Wang F., Song W., Zhang D., Lin W., Yin Q., Wang Q., Li H., Yuan Q., Zhang S. (2024). Magnesium promotes vascularization and osseointegration in diabetic states. Int. J. Oral Sci..

[50] Jing X., Wang S., Tang H., Li D., Zhou F., Xin L., He Q., Hu S., Zhang T., Chen T., Song J. (2022). Dynamically bioresponsive DNA hydrogel incorporated with dual-functional stem cells from apical papilla-derived exosomes promotes diabetic bone regeneration. ACS Appl. Mater. Interfaces.

[51] Khosla S., Samakkarnthai P., Monroe D.G., Farr J.N. (2021). Update on the pathogenesis and treatment of skeletal fragility in type 2 diabetes mellitus. Nat. Rev. Endocrinol..

[52] Claes L., Recknagel S., Ignatius A. (2012). Fracture healing under healthy and inflammatory conditions. Nat. Rev. Rheumatol..

[53] Mohsin S., Kaimala S., AlTamimi E.K.Y., Tariq S., Adeghate E. (2019). In vivo labeling of bone microdamage in an animal model of type 1 diabetes mellitus. Sci. Rep..

[54] Takanche J.S., Kim J.-E., Han S.-H., Yi H.-K. (2020). Effect of gomisin A on osteoblast differentiation in high glucose-mediated oxidative stress. Phytomedicine.

[55] Liu S., Lu M., Zhang M., Sun X., Luo B., Wu Y. (2025). Multifaceted catalytic glucose depletion and reactive oxygen species-scavenging nanoenzyme composite hydrogel for facilitating diabetic bone regeneration. ACS Nano.

[56] Wu B., Fu Z., Wang X., Zhou P., Yang Q., Jiang Y., Zhu D. (2022). A narrative review of diabetic bone disease: characteristics, pathogenesis, and treatment. Front. Endocrinol..

[57] Zhang X., Xu D., Zhang R., Wang H., Yang G. (2025). Interaction between diabetes and osteoporosis: imbalance between inflammation and bone remodeling. Osteoporos. Int..

