# Supplementary material for: Integrated apoptotic extracellular vesicle-recruitment peptide coating reprograms the diabetic bone microenvironment and orchestrates enhanced implant osseointegration
Source: Bioact Mater. 2026 Jun 11;65:484–505. doi: 10.1016/j.bioactmat.2026.05.059 (PMC13273899; doi:10.1016/j.bioactmat.2026.05.059)
Supplement: Multimedia component 1 [file mmc1.docx]

**Supplementary Information**

**Integrated Apoptotic Extracellular Vesicle-Recruitment Peptide Coating Reprograms the Diabetic Bone Microenvironment and Orchestrates Enhanced Implant Osseointegration**


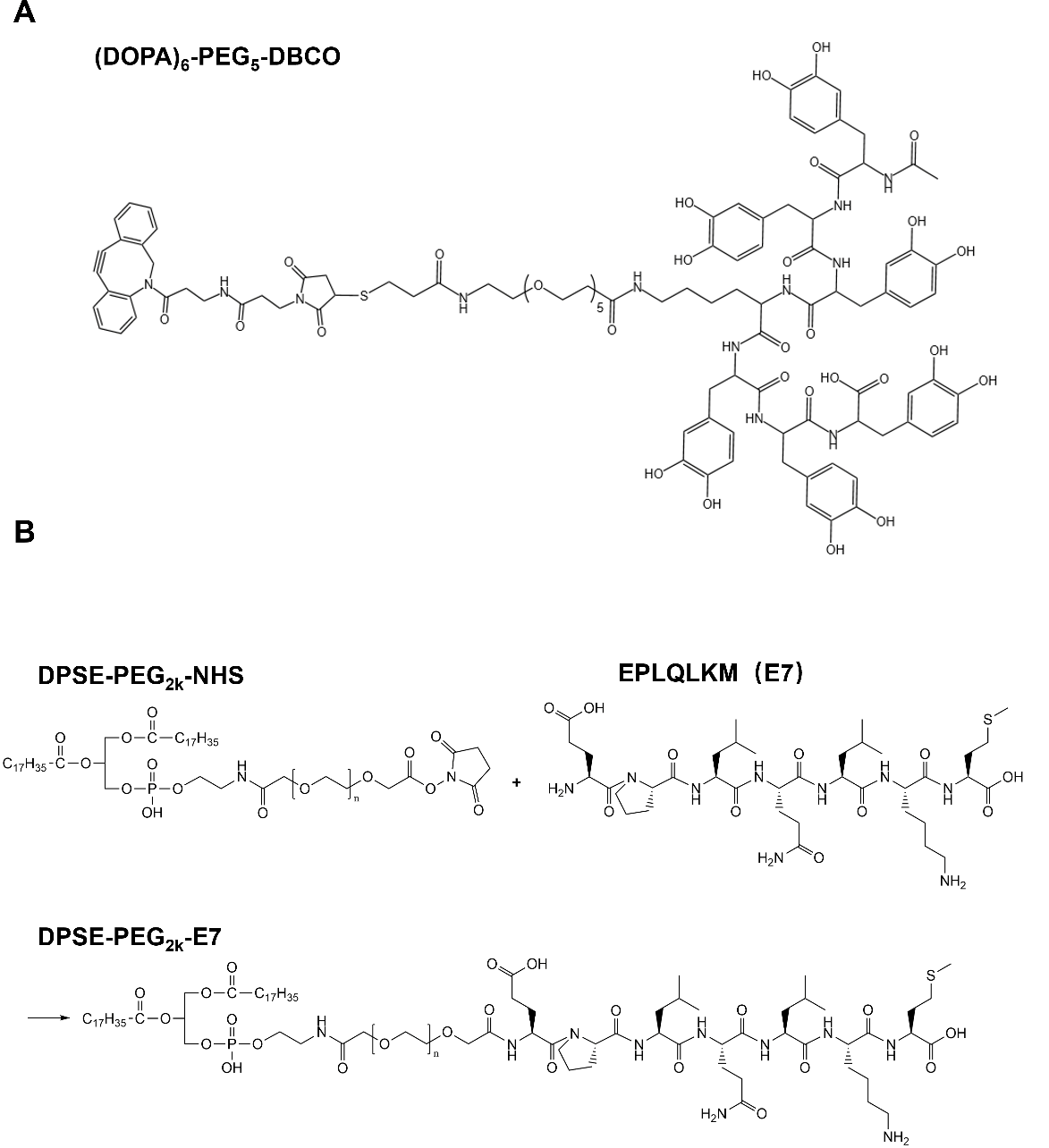


**Figure S1.** **Molecular Formula and Synthetic Process of the Peptide.** A) Molecular schematic and synthetic route of DSPE-PEG_2k_-EPLQLKM. B) Structural representation of the mussel-inspired peptide (DOPA)_6_-PEG_5_-DBCO.


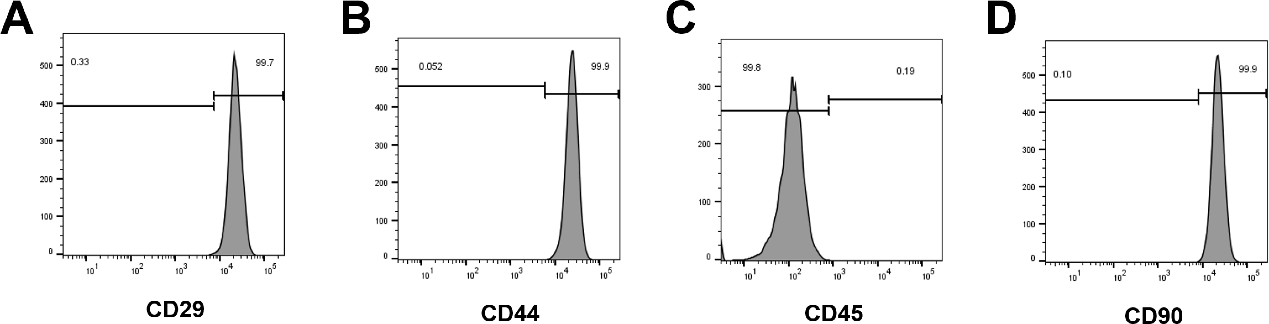


**Figure S2. Characterization of bone marrow-derived mesenchymal stem cells (BMSCs).** A–D) Flow cytometric analysis evaluating the expression of surface markers CD29, CD44, CD45, and CD90 in BMSCs.


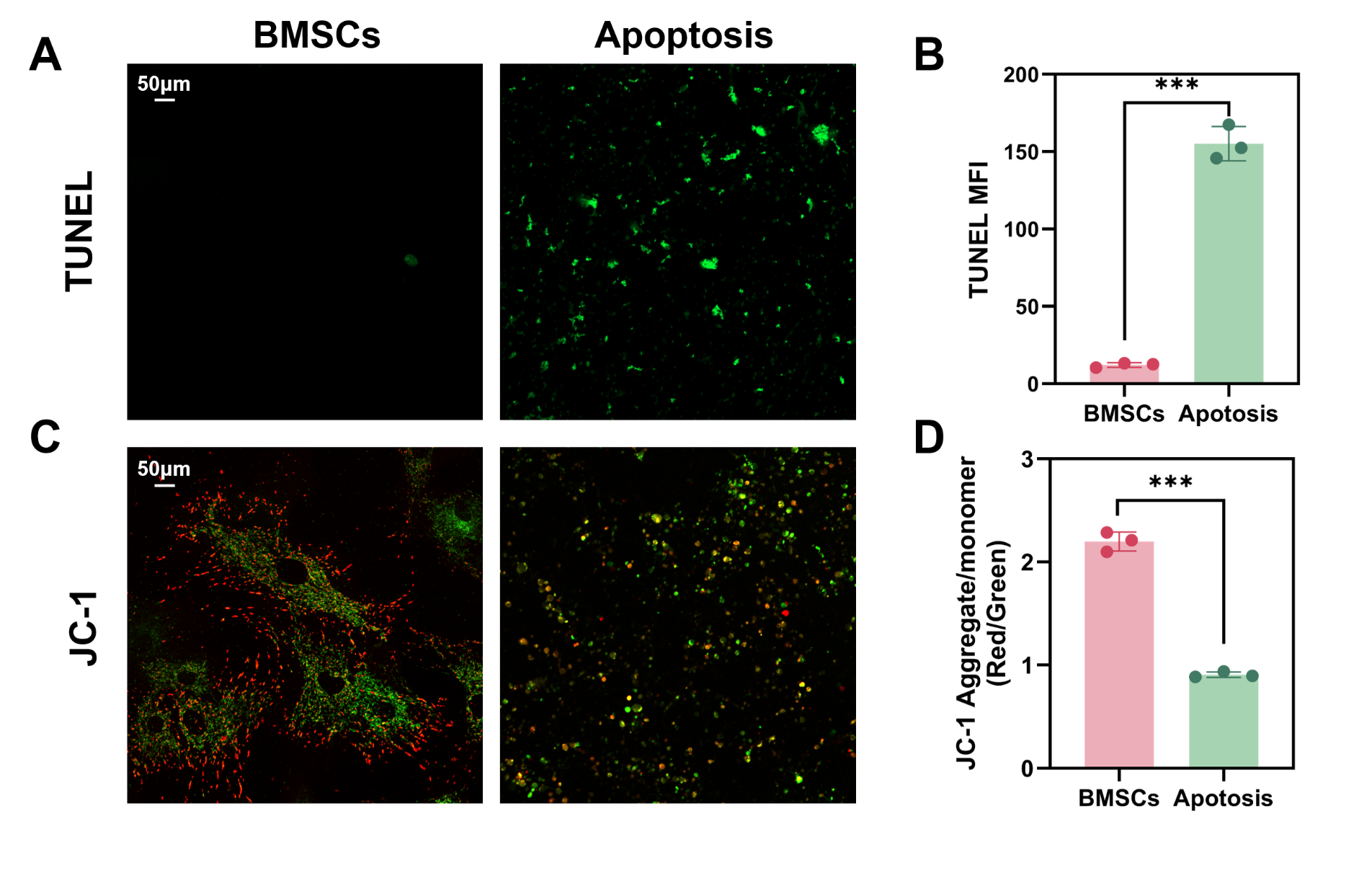


**Figure S3. Assessment of STS-induced apoptosis in BMSCs.** A, C) Evaluation of mitochondrial membrane potential changes following BMSC apoptosis via JC-1 staining, with corresponding quantitative analysis (scale bar = 50 μm, n=3).B, D) Evaluation of the apoptotic state of BMSCs using TUNEL assay and associated quantitative results (n=3).Data are presented as mean ± standard deviation (SD). statistical analysis was performed using Student's t-test. ***p < 0.001 indicates statistical significance.


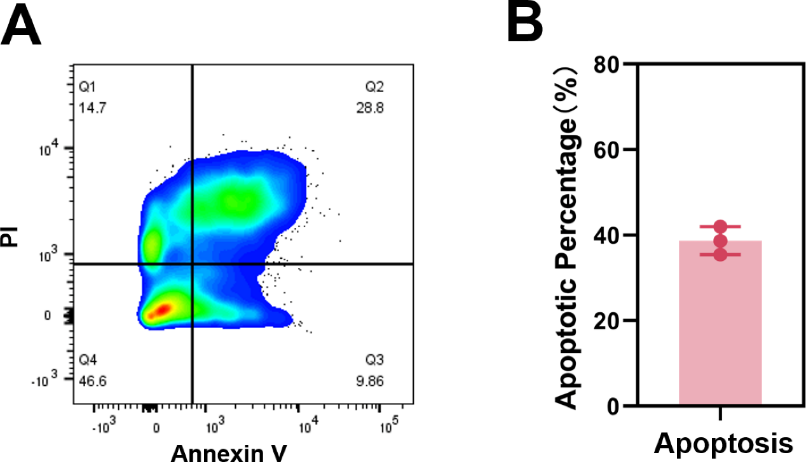


**Figure S4. STS-induced apoptosis in BMSCs.** A, B) Flow cytometric analysis of apoptotic BMSCs following STS induction using Annexin V-FITC/PI staining, with the corresponding calculation of apoptotic rates.


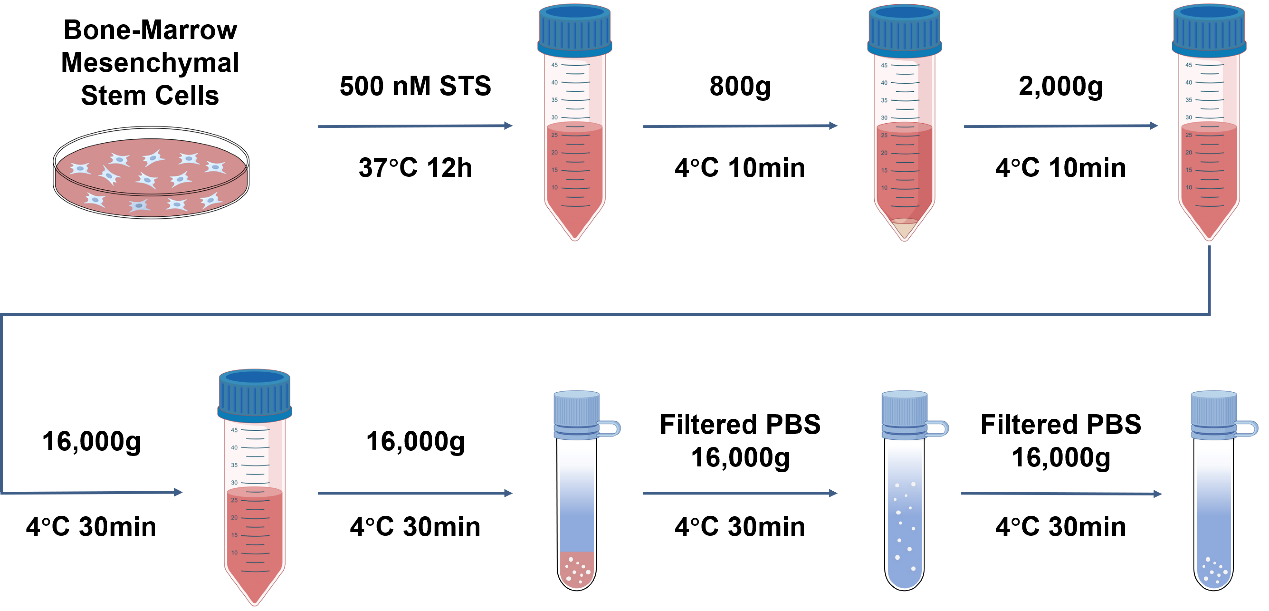


**Figure S5. Schematic workflow for the isolation of ApoEVs.**


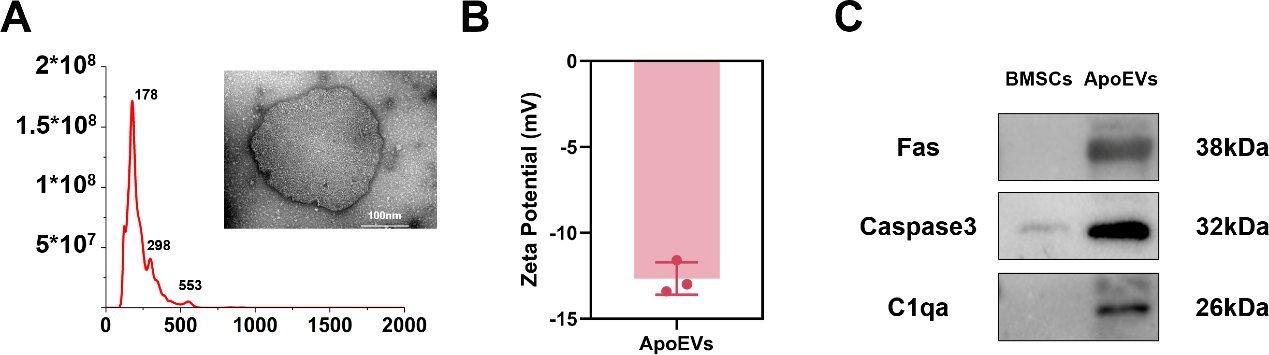


**Figure S6. Characterization of ApoEVs.** A) Particle size distribution of ApoEVs determined by NTA and morphological observation of spherical vesicles via TEM. B) Zeta potential of the isolated ApoEVs. C) Western blot analysis evaluating characteristic apoptotic markers in ApoEVs, including Fas, Caspase-3, and C1qa.

**
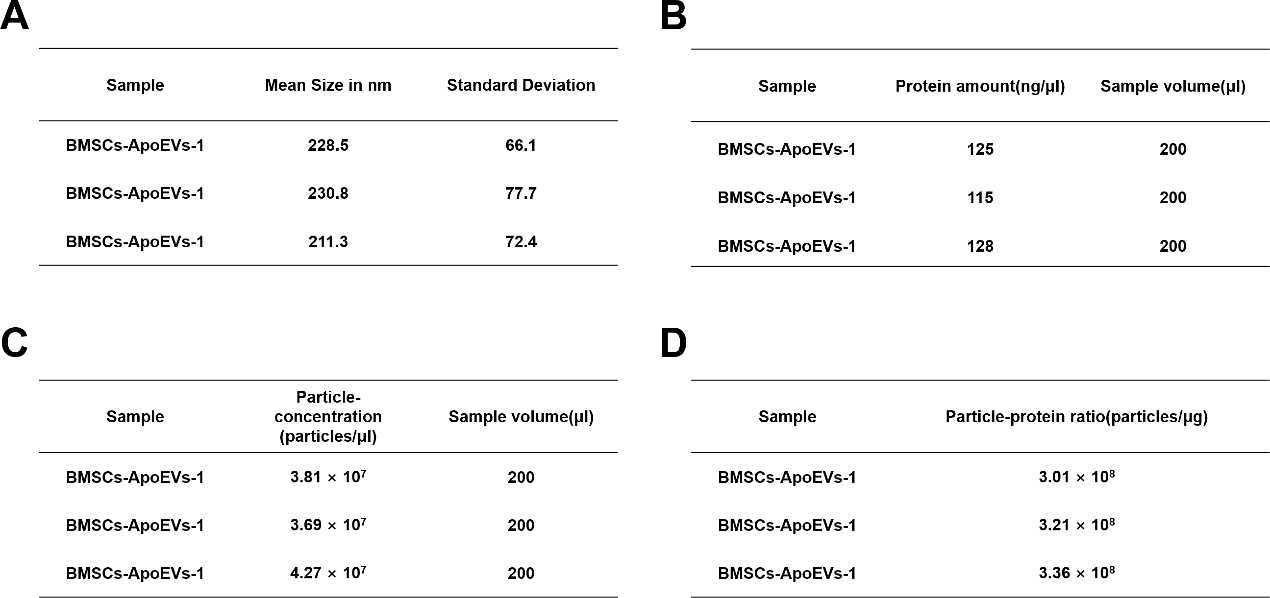
**

**Figure S7. Quantitative analysis of the isolated ApoEVs.** A) Size distribution of the isolated ApoEVs calculated via NTA. B) Quantification of protein content in the isolated ApoEVs using the BCA assay. C) Particle concentration of the isolated ApoEVs determined by NTA. D) Analysis of the particle/protein ratio in the ApoEVs samples.


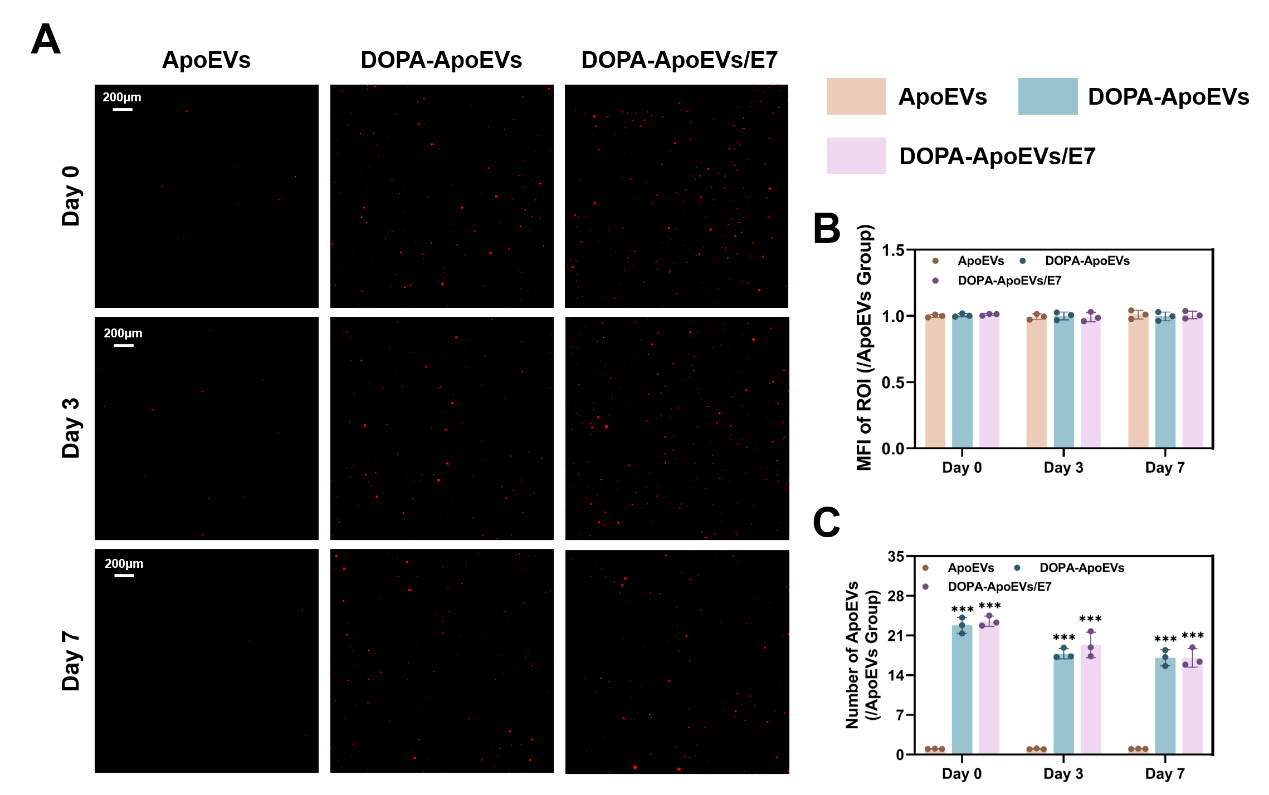


**Figure S8.** **Quantitative analysis of PKH26-labeled ApoEVs at different time points.** A) PKH26-labeled ApoEVs at days 0, 3, and 7. B–C) Statistical analysis of the mean fluorescence intensity (MFI) and total counts of the red fluorescently labeled ApoEVs (scale bar = 200 μm, n = 3). Data are expressed as mean ± standard deviation (SD), with statistical analysis performed using Two-way ANOVA and Tukey's post-hoc test. ***p < 0.001 indicate statistical significance.


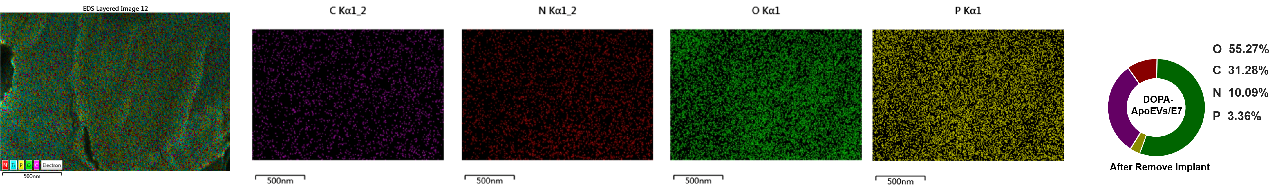


**Figure S9. SEM micrographs, EDS elemental mapping, and quantitative elemental composition analysis of the DOPA-ApoEVs/E7 peptide-modified surface.**


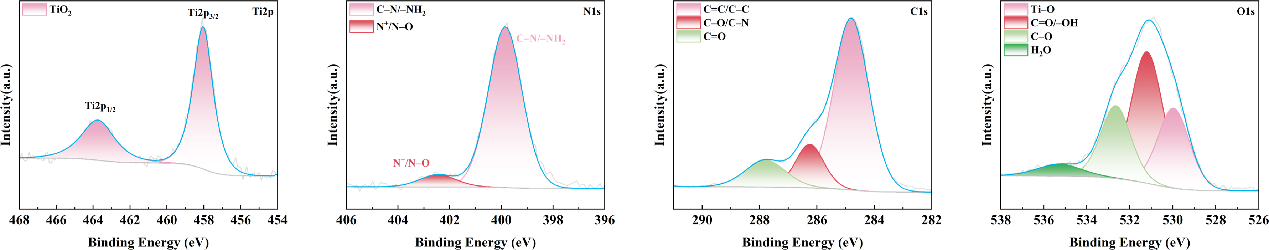


**Figure S10. Analysis of elemental fitted peaks in the XPS results of the DOPA-ApoEVs/E7 group**

**
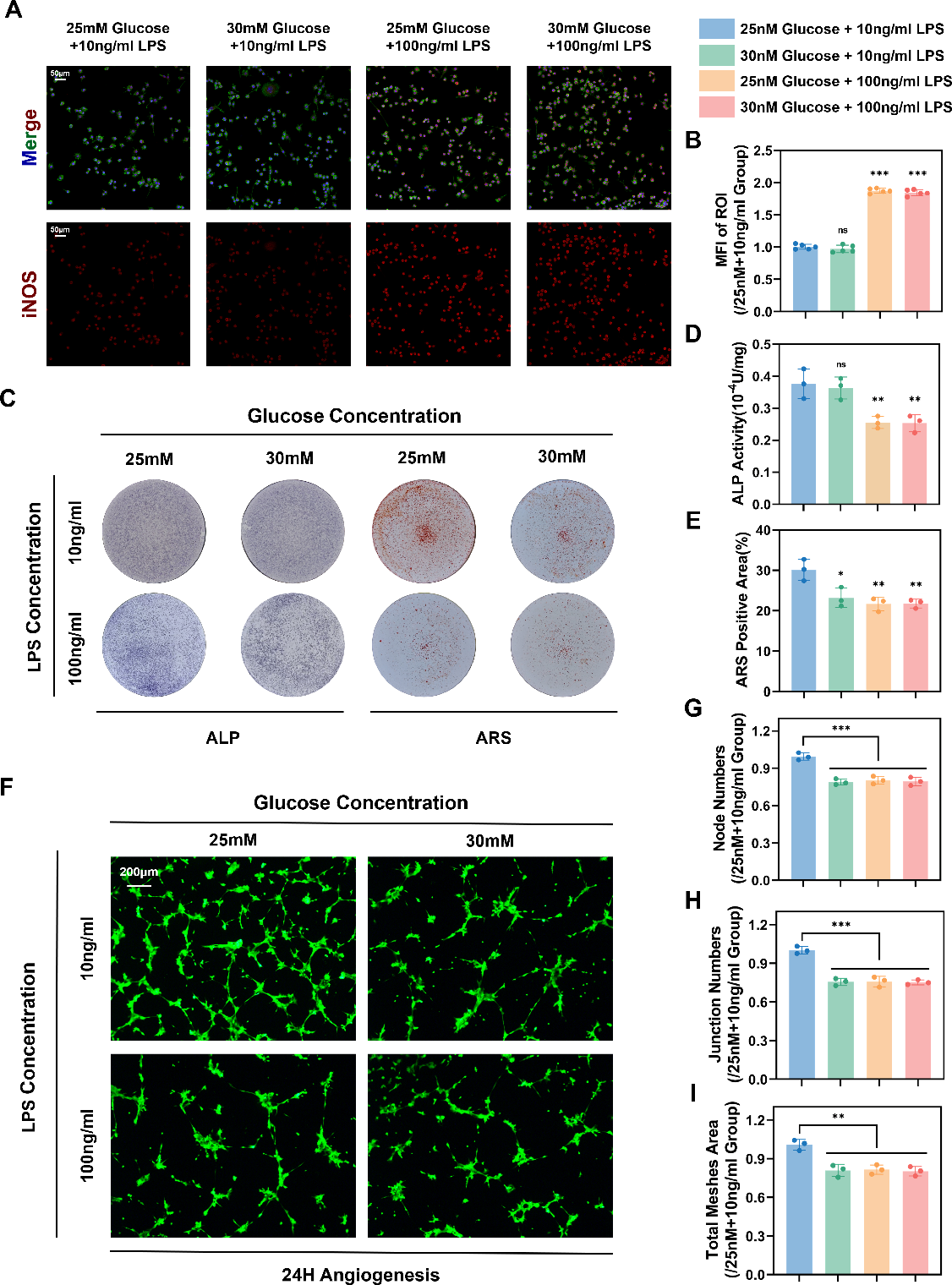
**

**Figure S11.** **Simulated diabetic microenvironment media with various formulation ratios.** A–B) Immunofluorescence staining and quantitative analysis evaluating macrophage M1 polarization; Green: cytoskeleton; Red: iNOS; Blue: nuclei (scale bar = 50 μm, n = 5). C–E) ALP and ARS assays and quantitative analysis evaluating osteogenic differentiation (n = 3). F–I) Evaluation and quantitative analysis of angiogenesis (scale bar = 200 μm, n = 3). Data are expressed as mean ± standard deviation (SD), with statistical analysis performed using one-way ANOVA and Tukey's post-hoc test. *p < 0.05, **p < 0.01, ***p < 0.001 indicate statistical significance and ns indicate no statistical significance.

**
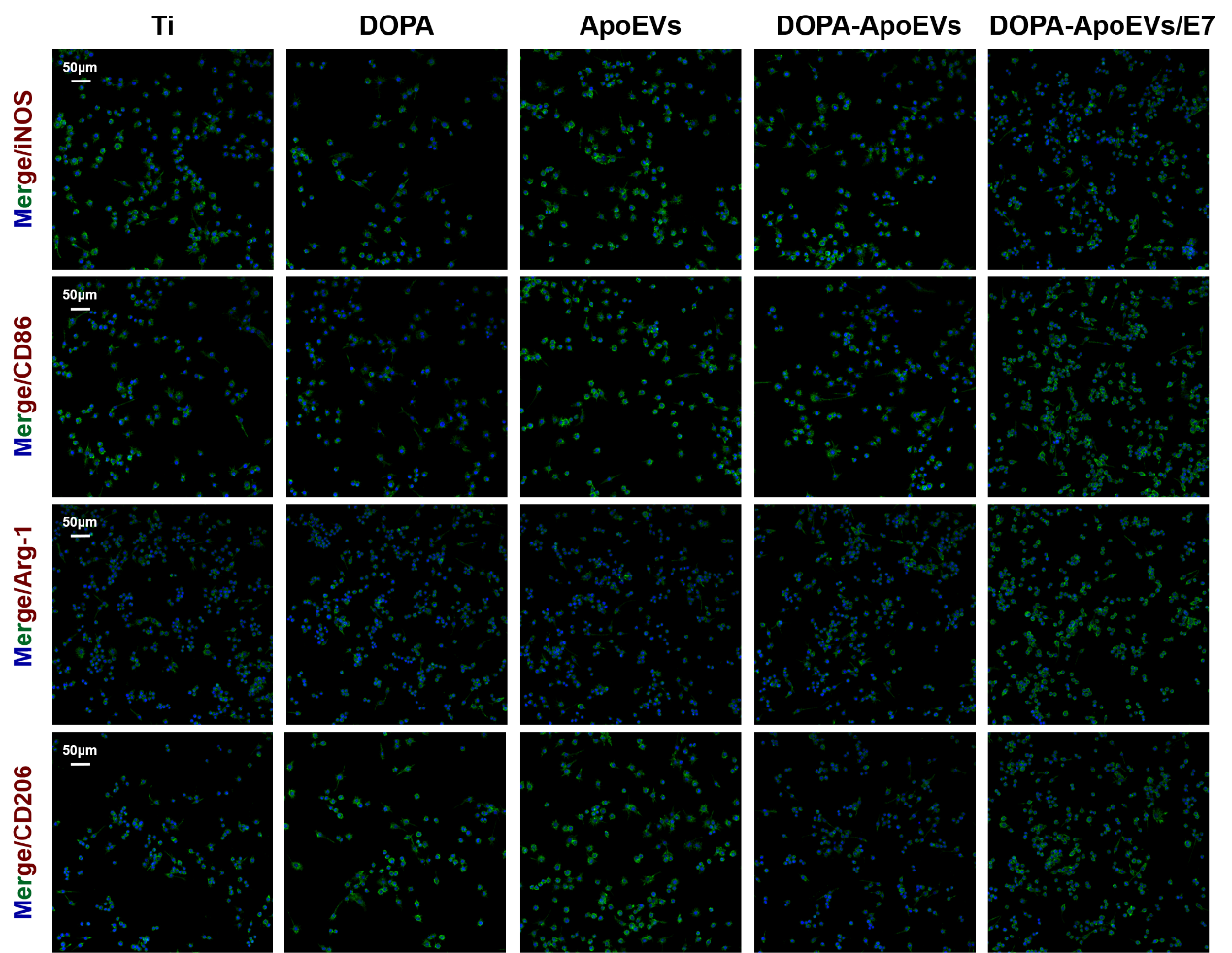
**

**Figure S12. Macrophage polarization in the non-diabetic microenvironment.** Immunofluorescence staining of macrophage polarization phenotypes. Green: cytoskeleton, Red: osteogenic markers (iNOS, CD86, Arg-1 or CD206), Blue: nuclei. (scale bar = 50 μm)

**
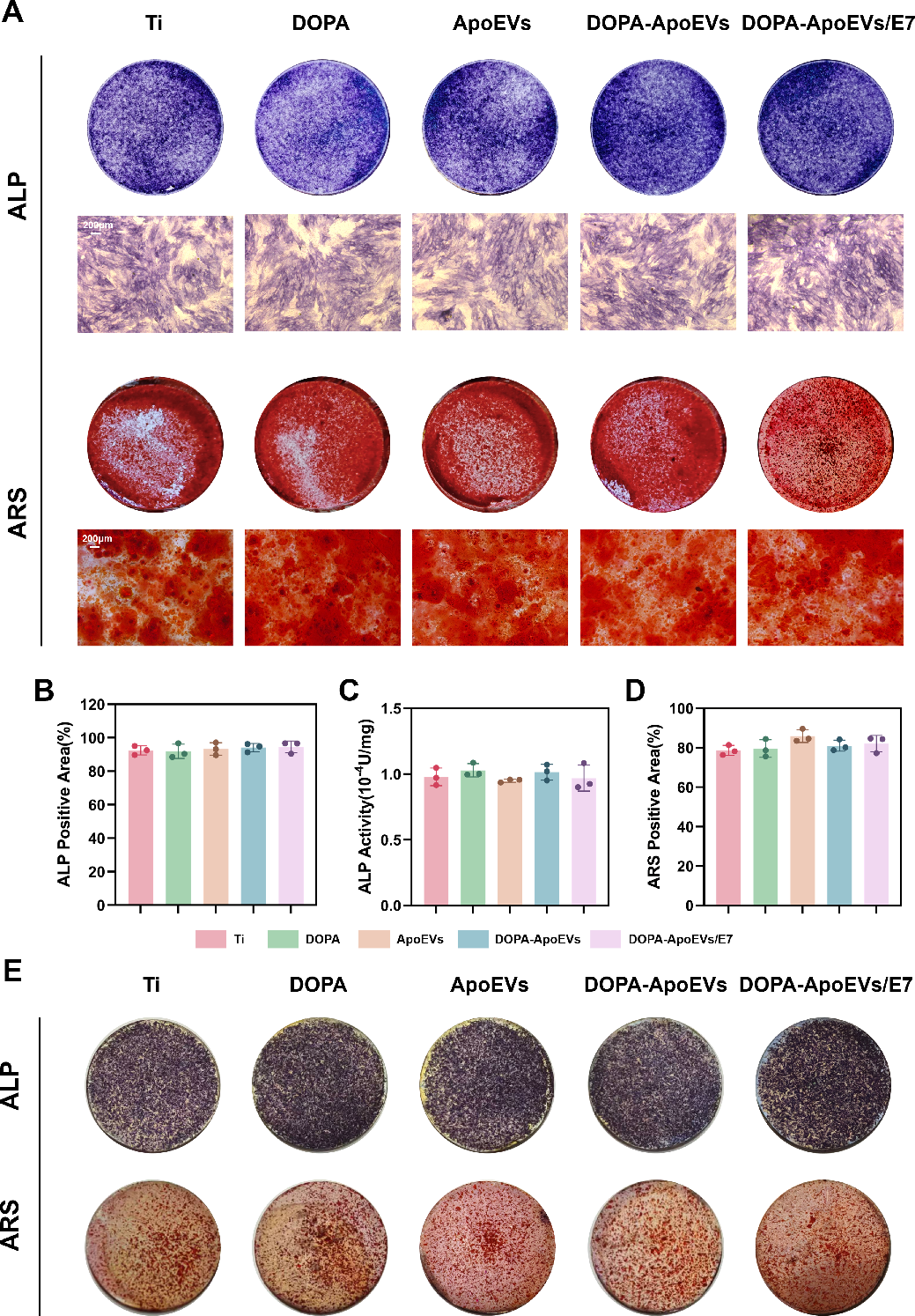
**

**Figure S13. Indirect and direct osteogenesis under the non-diabetic micro-environment.** A–D) BMSCs cultured in normal osteogenic induction medium under a non-diabetic environment. ALP and ARS staining were performed to evaluate osteogenic differentiation, followed by the corresponding quantitative analysis (scale bar = 200 μm, n = 3). E) BMSCs seeded directly onto Titanium (Ti) discs with different modified surfaces and cultured in normal medium; ALP and ARS assays were utilized to evaluate the osteogenic effects in comparison with the diabetic groups. Data are expressed as mean ± standard deviation (SD), with statistical analysis performed using one-way ANOVA and Tukey's post-hoc test, ns indicate no statistical significance.


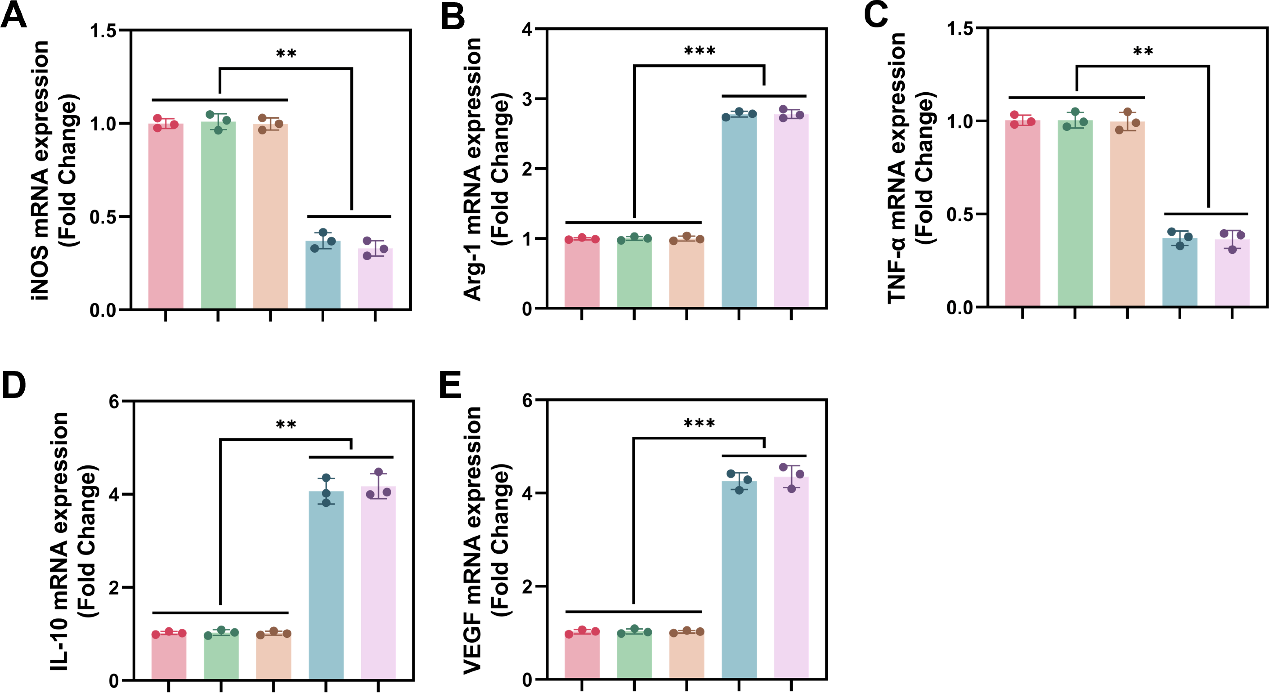


**Figure S14. Gene expression profiles of DOPA-ApoEVs modified surfaces under DM in vitro conditions.** A, B) RT-qPCR assessment of the M1 macrophage marker iNOS and M2 macrophage marker Arg-1 at the mRNA level (n = 3). C, D) RT-qPCR evaluation of the pro-inflammatory marker TNFα and anti-inflammatory marker IL-10 gene expression (n = 3). E) RT-qPCR analysis of the angiogenic marker VEGF gene expression (n = 3). Data are expressed as mean ± standard deviation (SD), with statistical analysis performed using one-way ANOVA and Tukey's post-hoc test. **p < 0.01, and ***p < 0.001 indicate statistical significance.


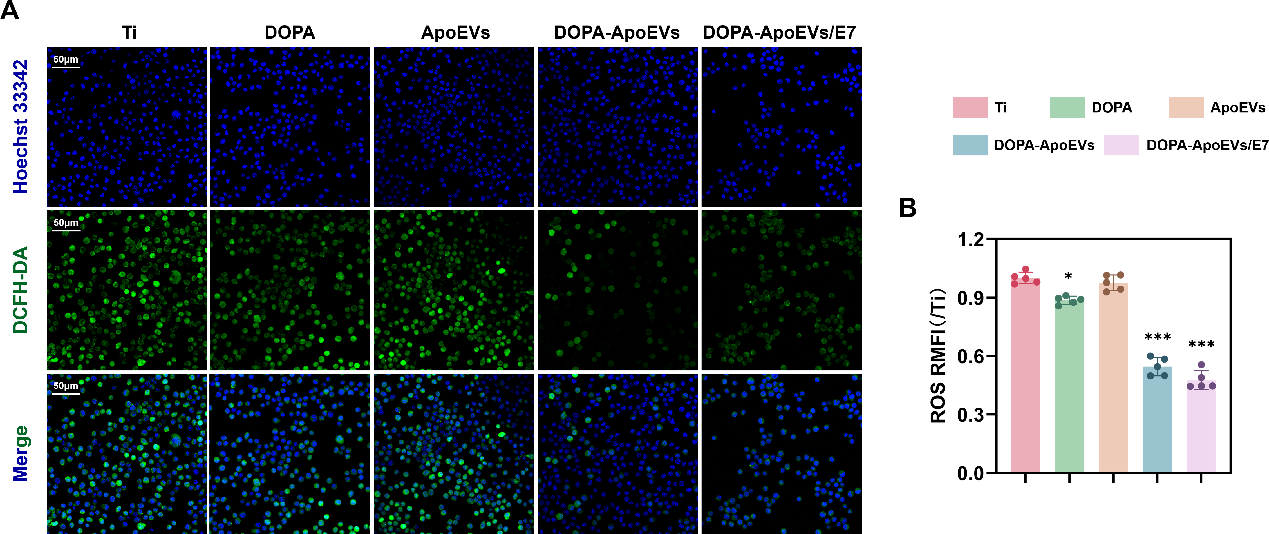


**Figure S15. ROS Fluorescent Probe Staining and Quantitative Analysis.** A) ROS Fluorescent Probe Staining. Green: DCFH-DA, Blue: Hoechst 33342(scale bar = 200 μm, n = 5). B) Quantitative analysis of fluorescence intensity. Data are expressed as mean ± standard deviation (SD), with statistical analysis performed using one-way ANOVA and Tukey's post-hoc test. *p < 0.05, and ***p < 0.001 indicate statistical significance.


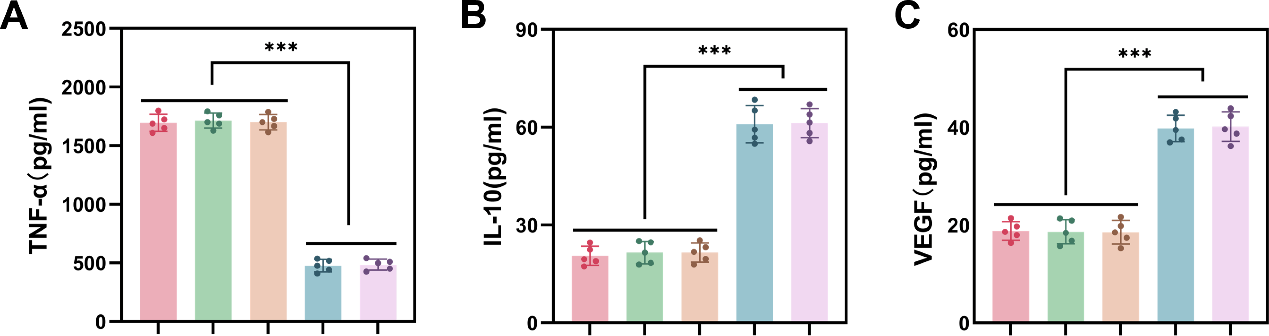


**Figure S16. Regulation of cytokine secretion by DOPA-ApoEVs modified surfaces under DM in vitro conditions.** ELISA quantification of pro-inflammatory cytokine TNF-α, anti-inflammatory cytokine IL-10, and VEGF secretion levels (n=5). Data are expressed as mean ± standard deviation (SD), with statistical analysis performed using one-way ANOVA and Tukey's post-hoc test. ***p < 0.001 indicate statistical significance.


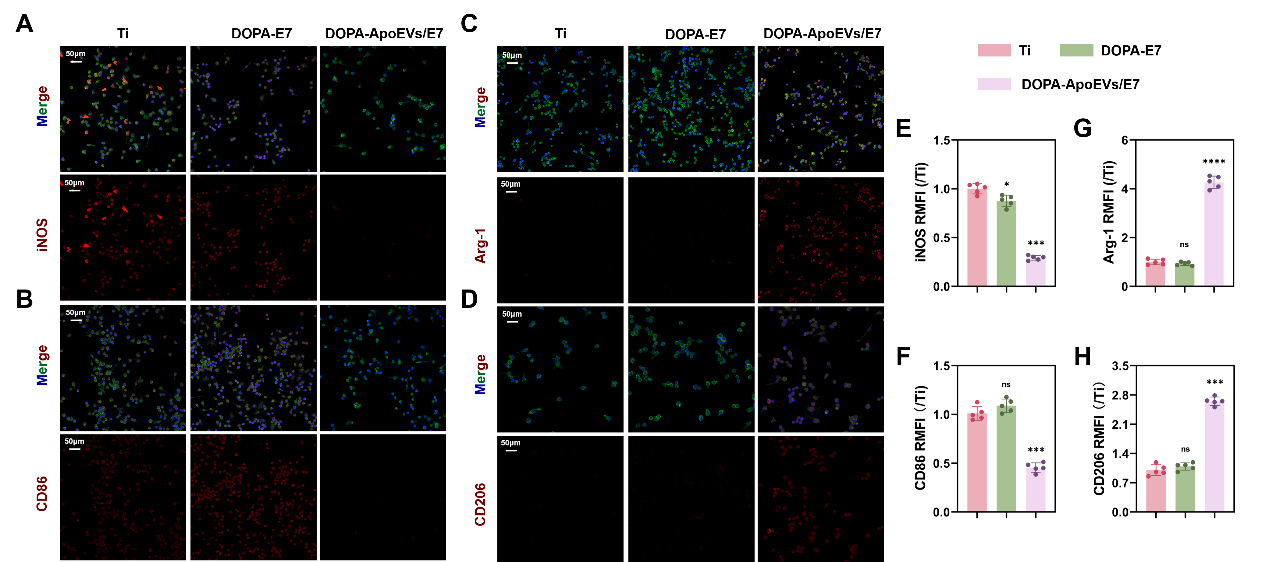


**Figure S17. Macrophage polarization in the DOPA-E7 group.** A–D) Immuno-fluorescence staining of macrophage polarization phenotypes. Green: cytoskeleton, Red: osteogenic markers (iNOS, CD86, Arg-1 or CD206), Blue: nuclei. E–H) Quantitative analysis of marker protein expressions for M1 and M2 phenotypes((scale bar = 50 μm, n=5). Data are expressed as the mean ± standard deviation (SD), with statistical analysis performed using one-way ANOVA and Tukey's post-hoc test. *p < 0.05, ***p < 0.001 and ****p < 0.0001 indicate statistical significance and ns indicate no statistical significance.

**
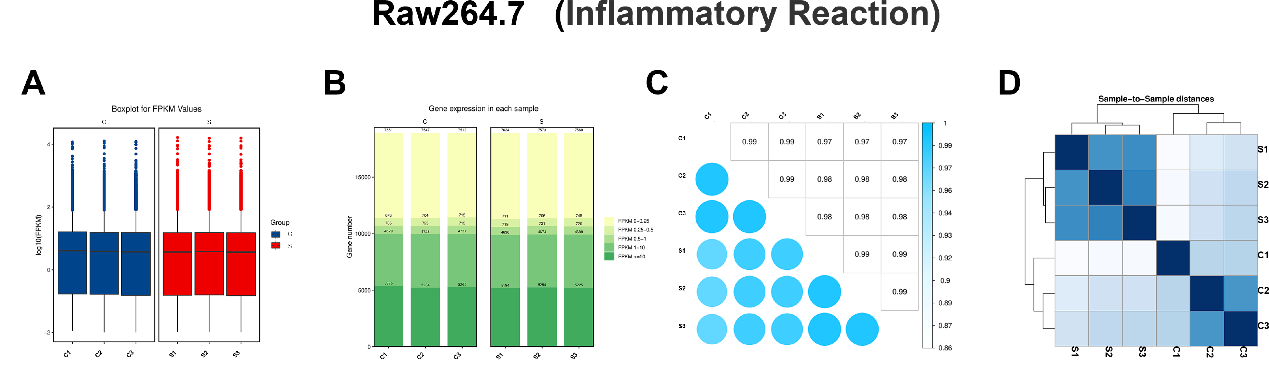
**

**Figure S18. Supplementary quality control (QC) data for transcriptomic sequencing results.** A) Box plot of gene expression levels. B) Density distribution plot of gene expression levels. C) Heatmap showing the correlation coefficients between samples. D) Dendrogram representing the results of hierarchical clustering analysis among samples. Statistical analysis was performed using the unpaired two-tailed t-test.

**
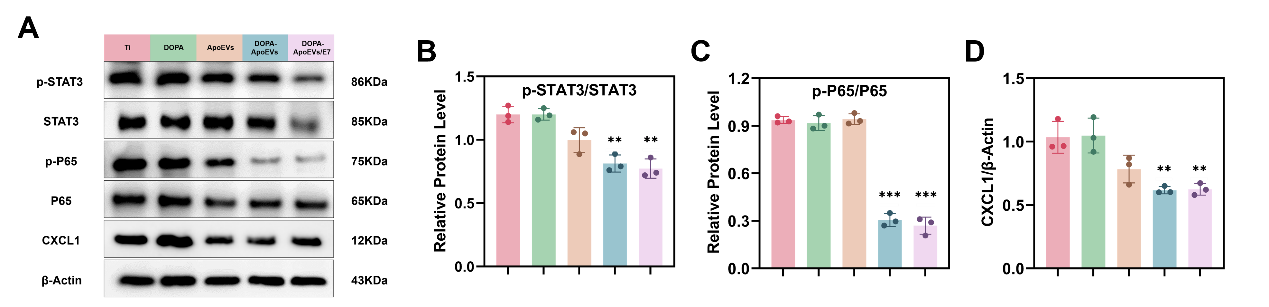
**

**Figure S19. DOPA-ApoEVs/E7 regulates inflammation-related pathways.** Nf-κB, JAK-STAT pathway and CXCL1 activated protein western blot detection. Data are expressed as mean ± standard deviation (SD), with statistical analysis performed using one-way ANOVA and Tukey's post-hoc test.***p < 0.001 indicate statistical significance.


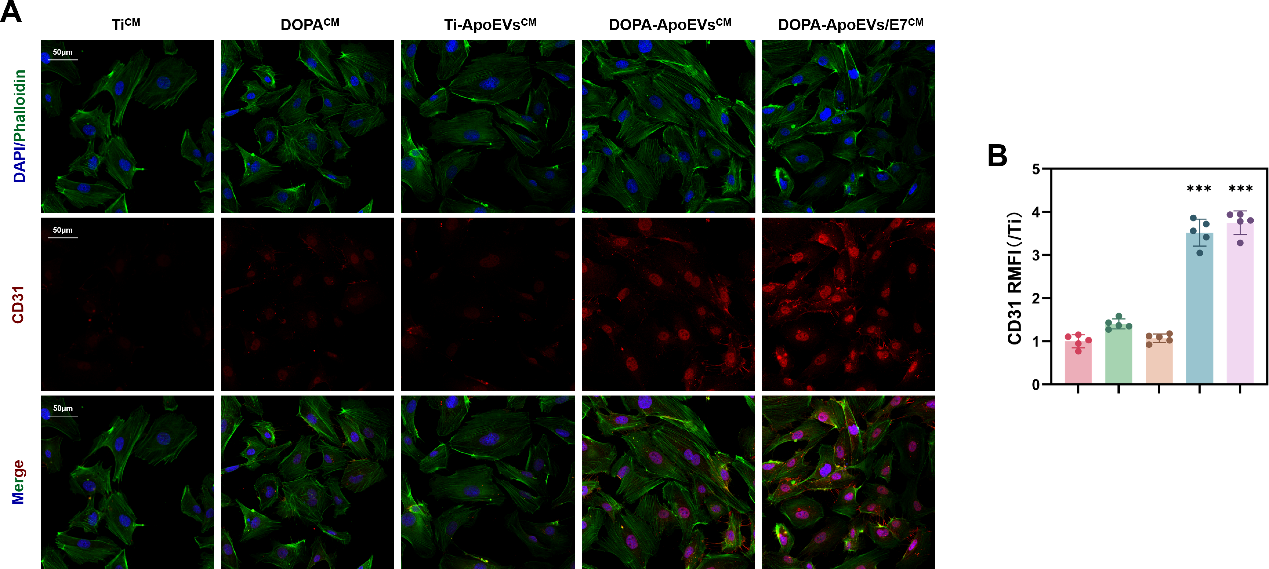


**Figure S20. DOPA-ApoEVs modified surfaces promote angiogenesis under DM inflammatory conditions.** A, B) Immunofluorescence staining evaluating VEGF expression alongside the corresponding quantitative analysis (scale bar = 50 μm, n = 3). Data are expressed as mean ± standard deviation (SD), with statistical analysis performed using one-way ANOVA and Tukey's post-hoc test. ***p < 0.001 indicate statistical significance.


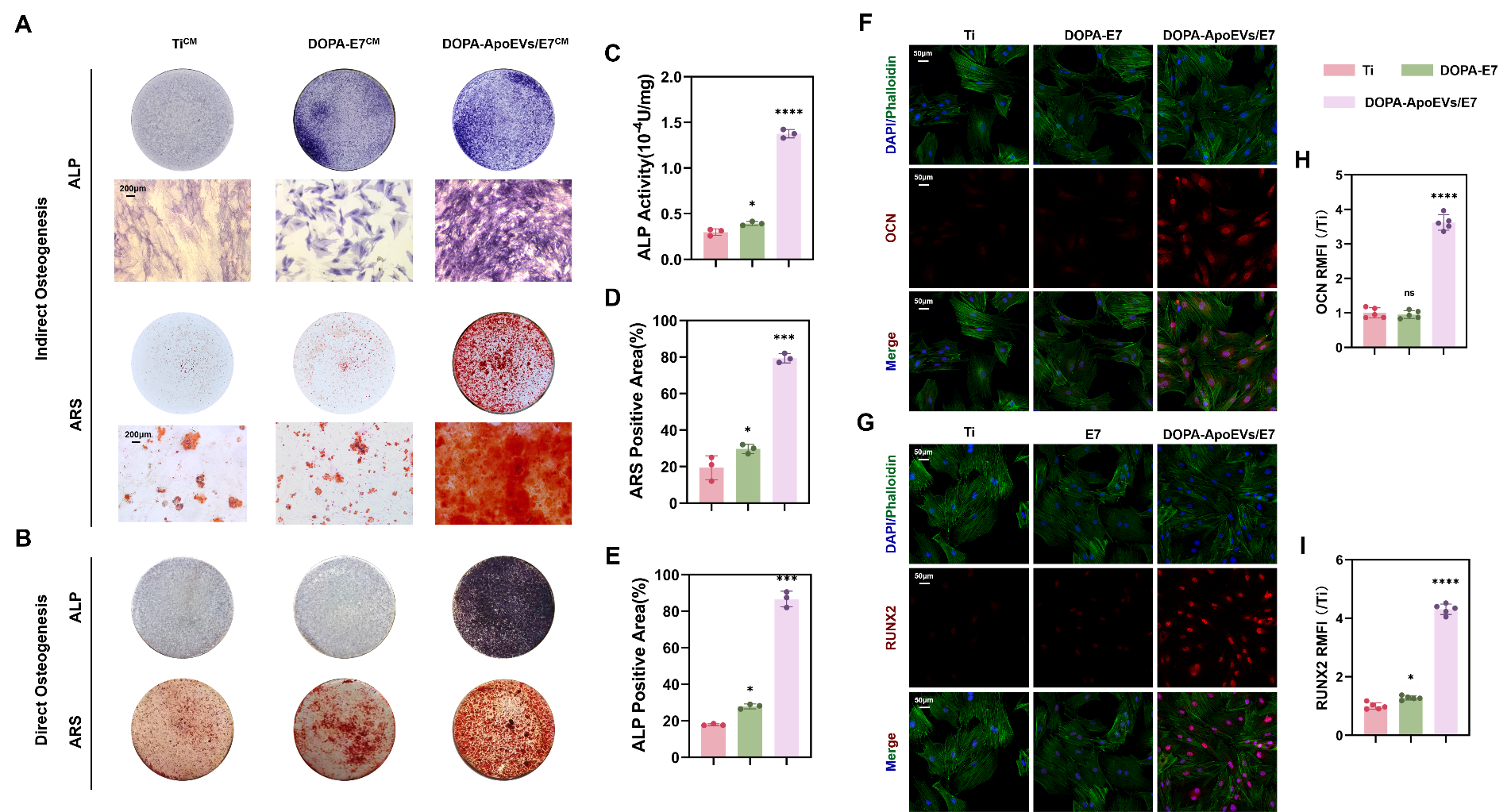


**Figure 21. E7-modified surfaces promote direct and indirect osteogenesis under DM inflammatory conditions.** A–E) Bone marrow-derived mesenchymal stem cells (BMSCs) cultured in conditioned medium supplemented with osteogenic induction factors; Compared with the Ti group, ALP and ARS staining were performed to evaluate osteogenesis, followed by corresponding quantitative analysis(scale bar = 200 μm, n = 3). (F–I) Immunofluorescence staining and quantitative analysis of osteogenic hallmark proteins RUNX2 and OCN compared with the Ti group in BMSCs; Green: cytoskeleton, Red: osteogenic markers (RUNX2 or OCN), Blue: nuclei (scale bar = 200 μm, n = 5). Data are expressed as the mean ± standard deviation (SD), with statistical analysis performed using one-way ANOVA and Tukey's post-hoc test. *p < 0.05, **p < 0.01, ***p < 0.001 and ****p < 0.0001 indicate statistical significance.

**
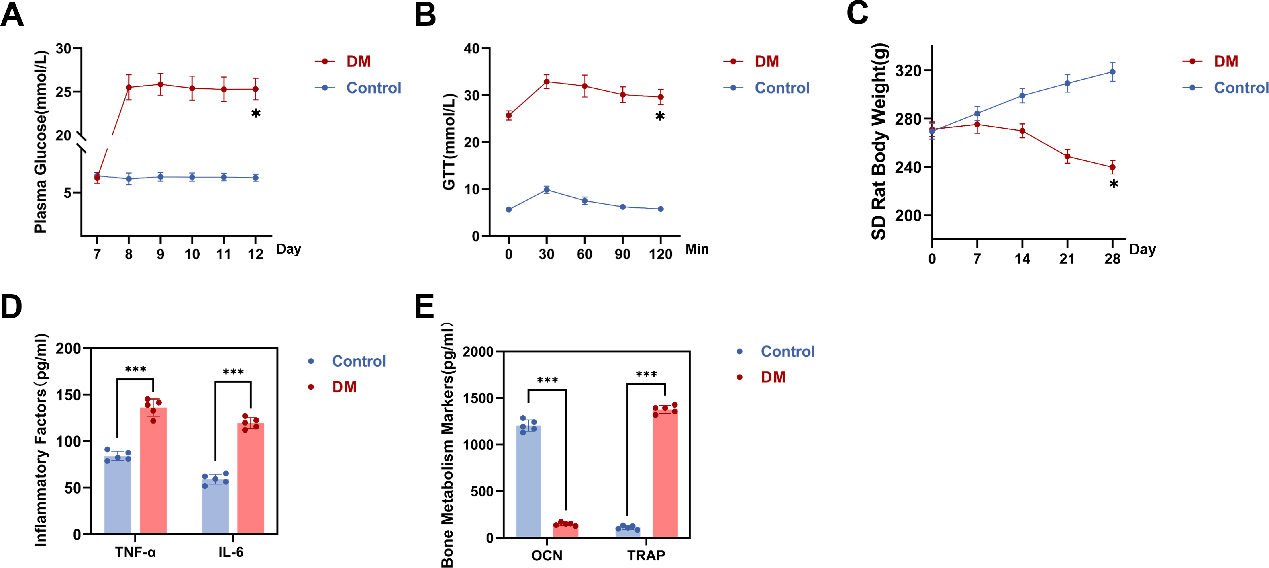
****Figure S22. Validation of the STZ-Induced Diabetic Rat Model.** A) Fasting blood glucose monitoring of SD rats following DM model induction. B) Glucose Tolerance Test (GTT) results post-DM modeling. C) Body weight of DM rats. D) Serum inflammatory cytokines of DM rats. E) Serum bone metabolism markers of DM rats. Data are expressed as mean ± standard deviation (SD), with statistical analysis performed using Student's t-test. *p < 0.05 and ***p < 0.001 indicate statistical significance.


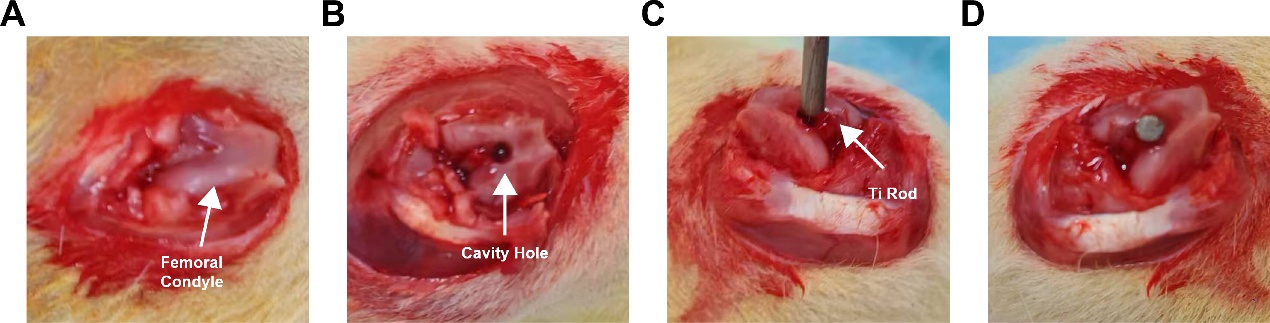


**Figure S23. Surgical procedure for titanium rod implantation into the rat femur.** A) Exposure of the femoral condyle. B) Drilling of the femoral condyle. C) Implantation of the titanium rod. D) Completion of the titanium rod implantation.


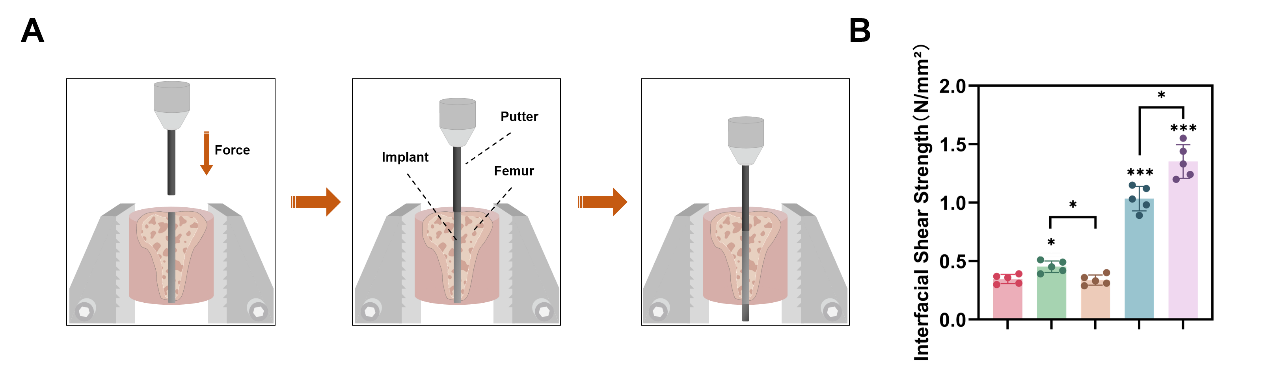


**Figure S24. Biomechanical Evaluation of Implants.** A) Schematic illustration of the maximum push-out force test for the implanted titanium rods. B) Statistical analysis of interfacial shear strength(n=5). Data are expressed as mean ± standard deviation (SD), with statistical analysis performed using one-way ANOVA and Tukey's post-hoc test. *p < 0.05 and *p < 0.001 indicate statistically significant.

**
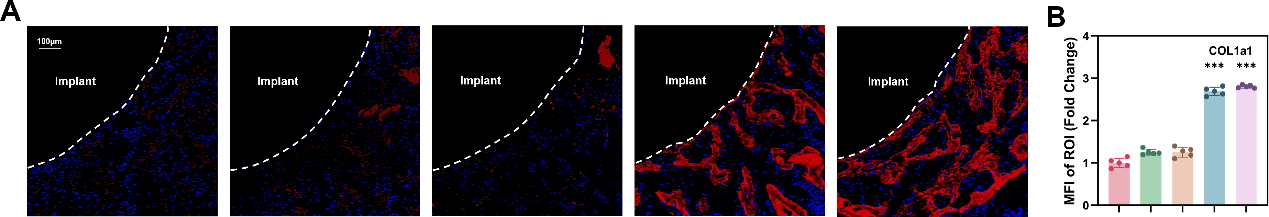
Figure S25. DOPA-ApoEVs/E7 modified surfaces enhance in vivo osseointegration under DM conditions.** A, B) Immunofluorescence staining and quantitative analysis of the osteogenic marker Col1a1 in BMSCs; red: osteogenic markers (Col1a1), blue: nuclei (scale bar = 100 μm, n=5). Data are expressed as mean ± standard deviation (SD), with statistical analysis performed using one-way ANOVA and Tukey's post-hoc test. ***p < 0.001 indicate statistical significance.

**Tables**


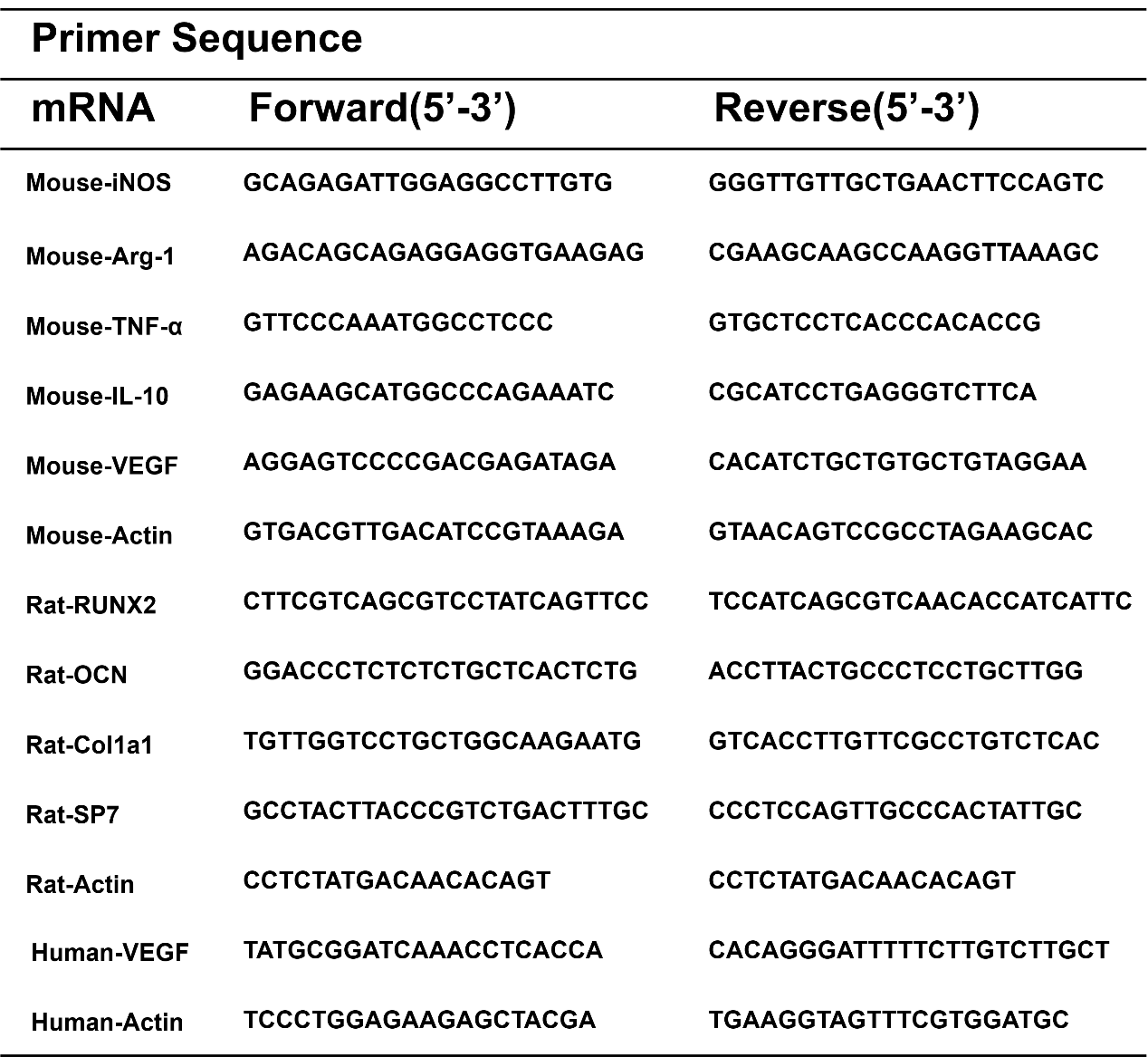


**Table S1. Primer sequence**
